# A regulatory circuit of miR-125b/miR-20b and Wnt signalling controls glioblastoma phenotypes through FZD6-modulated pathways

**DOI:** 10.1038/ncomms12885

**Published:** 2016-10-04

**Authors:** Tianzhi Huang, Angel A. Alvarez, Rajendra P. Pangeni, Craig M. Horbinski, Songjian Lu, Sung-Hak Kim, C. David James, Jeffery J. Raizer, John A. Kessler, Cameron W. Brenann, Erik P. Sulman, Gaetano Finocchiaro, Ming Tan, Ryo Nishikawa, Xinghua Lu, Ichiro Nakano, Bo Hu, Shi-Yuan Cheng

**Affiliations:** 1 Department of Neurology, Northwestern Brain Tumor Institute, The Robert H. Lurie Comprehensive Cancer Center, Northwestern University Feinberg School of Medicine, Chicago, Illinois 60611, USA; 2 Department of Neurological Surgery, Northwestern Brain Tumor Institute, The Robert H. Lurie Comprehensive Cancer Center, Northwestern University Feinberg School of Medicine, Chicago, Illinois 60611, USA; 3 Department of Biomedical Informatics, University of Pittsburgh, Pittsburgh, Pennsylvania 15206, USA; 4 Department of Neurosurgery, University of Alabama at Birmingham, Birmingham, Alabama 35294, USA; 5 Human Oncology and Pathogenesis Program, Department of Neurosurgery, Brain Tumor Center, Memorial Sloan-Kettering Cancer Center, New York, New York 10065, USA; 6 Department of Radiation Oncology, The University of Texas M.D. Anderson Cancer Center, 1515 Holcombe Boulevard, Houston, Texas 77030, USA; 7 Unit of Molecular Neuro-Oncology, Department of Neuro-Oncology, Fondazione IRCCS Istituto Neurologico, Via Celoria 11, 20133 Milano, Italy; 8 Mitchell Cancer Institute, University of South Alabama, Mobile, Alabama 36604, USA; 9 Department of Neuro-Oncology/Neurosurgery, Saitama Medical University International Medical Center, Saitama, 350-1298, Japan

## Abstract

Molecularly defined subclassification is associated with phenotypic malignancy of glioblastoma (GBM). However, current understanding of the molecular basis of subclass conversion that is often involved in GBM recurrence remain rudimentary at best. Here we report that canonical Wnt signalling that is active in proneural (PN) but inactive in mesenchymal (MES) GBM, along with miR-125b and miR-20b that are expressed at high levels in PN compared with MES GBM, comprise a regulatory circuit involving TCF4-miR-125b/miR-20b-FZD6. FZD6 acts as a negative regulator of this circuit by activating CaMKII–TAK1–NLK signalling, which, in turn, attenuates Wnt pathway activity while promoting STAT3 and NF-κB signalling that are important regulators of the MES-associated phenotype. These findings are confirmed by targeting differentially enriched pathways in PN versus MES GBM that results in inhibition of distinct GBM subtypes. Correlative expressions of the components of this circuit are prognostic relevant for clinical GBM. Our findings provide insights for understanding GBM pathogenesis and for improving treatment of GBM.

## Abstract (web summary)

Glioblastoma (GBM) is classified as proneural (PN), neural, mesenchymal (MES) and classical GBM. Here the authors show that Wnt signalling, miR-125b and miR-20b establish a regulatory circuitry including FZD6 which distinguishes PN from the MES subtype.

## Graphical abstract

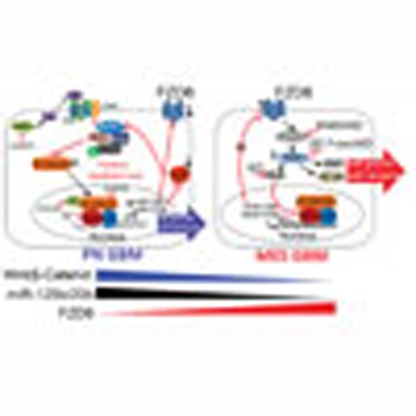

Glioblastoma (GBM) is the most common primary malignant brain tumour^1^. Integrated genomic analyses by The Cancer Genome Atlas (TCGA) Network revealed that GBM can be subclassified into three or four clinically relevant types: proneural (PN), neural, mesenchymal (MES) and classical GBM, each characterized by a distinct gene expression signature, as well as by specific genetic alterations^2^,^3^,^4^. Studies have shown that GBM with MES-associated gene expression signatures have worse prognosis compared with those with a PN subtype^4^,^5^. Glioma stem cells (GSCs) are tumour cell subpopulations in GBM that have stem cell-like properties, and are considered important contributors to GBM aggressiveness, recurrence and therapy resistance^6^. GSCs that grow as tumour spheres *in vitro* retain the characteristics of the original tumour and can be stratified according to tumour subtype^7^,^8^. PN and MES GSCs (henceforth referred as to glioma spheres) have distinct dysregulated signalling pathway ‘signatures' that contribute to their malignant phenotypes and response to radiation^9^. The conversion of PN to MES subtype in GBM has been reported by others, and usually in association with tumour recurrence following initial treatment^10^,^11^,^12^. Currently, there is substantial interest in determining whether GBM subtype-defining mutations, expression patterns, and signalling pathway dysregulation predict tumour response and adaptation to targeted therapies^13^,^14^.

MicroRNAs (miRs) are small noncoding RNAs that act as modulators of gene expression in all multicellular organisms. A unique feature of miRs is that a single miR can simultaneously regulate the expression of multiple target genes, thereby affecting numerous cellular behaviours including differentiation, proliferation, and survival^15^. Dysregulated miR expression plays an important role in cancer^16^,^17^. Distinct miR expression patterns have been described in cancer stem cells (CSCs) from individual tumours, including GBM, suggesting cancer cell type-specific miR functions^18^. Furthermore, for GBM, there are onco-miR clusters that influence survivorship^19^, underscoring the effects of miRs on tumour heterogeneity.

Wnt signalling is involved in embryonic development, adult tissue self-renewal, tissue repair, and cancer^20^. Canonical Wnt signalling is mediated from Frizzled (FZD) family receptors to β-catenin, which acts with transcription factors to induce the expression of genes that regulate differentiation and self-renewal, such as *LEF1*, *SOX2*, *JAG1* and *ID2* (ref. 21). Positive regulators of Wnt signalling include co-receptors low-density lipoprotein receptor-related protein 5 and 6 (LRP5/6), and Wnt ligand WNT3a, whereas negative regulators include FZD6, APC, AXIN and GSK3β. Canonical Wnt signalling regulates phenotypes of normal and CSCs, whereas non-canonical Wnt signalling controls cell movement and polarity^22^,^23^. In addition, several miRs have been shown to influence tumorigenesis by affecting the Wnt pathway activity^24^,^25^. In this study, we report that miR-125b and miR-20b are key mediators of the Wnt activity that are active in PN, but not in MES GBM. miR-125b, miR-20b and the Wnt pathway establish a regulatory circuit that includes FZD6 as the key negative regulator of the Wnt signalling. Due to the GBM subtype specificity of Wnt signalling, our results have important clinical implications for the development of targeted therapies against specific tumour subtypes.

## Results

### Expression of miRs distinguishes PN from MES GBM

**Figure 1:**
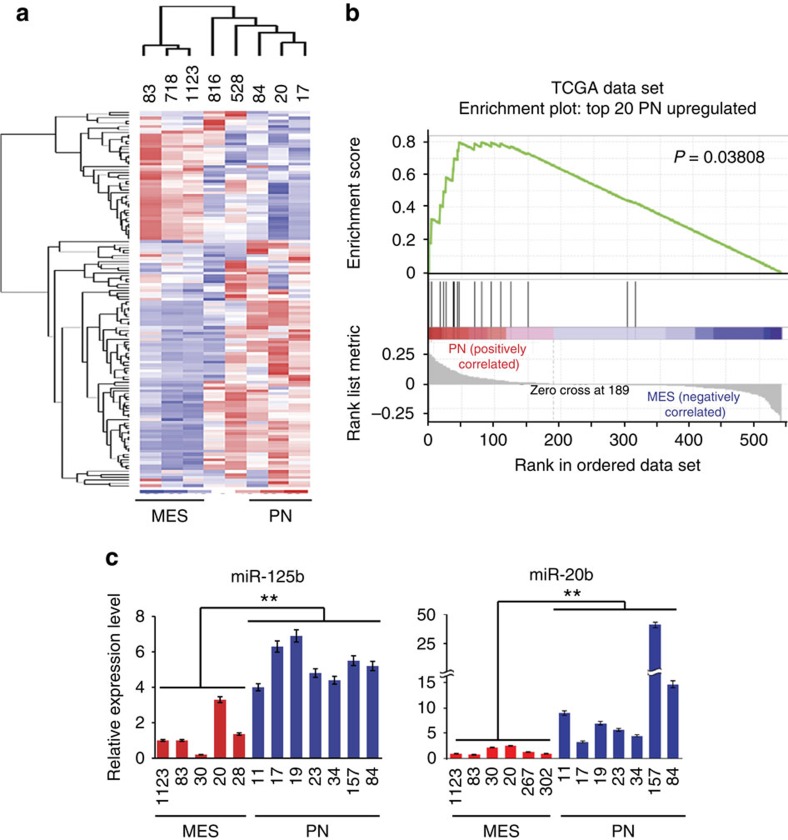
miR profiling and expressions of miR-125b and miR-20b distinguish PN and MES subtypes in GBM. (**a**) miRs are differentially expressed in PN 17, 20, and 84, MES 1123, 83, and 528, 718, and 816 glioma spheres. Heat maps were generated from the data of Nanostring nCounter miR Arrays using Cluster 3.0 and Java Treeview 1.1.6. (**b**) Gene Set Enrichment Analysis (GSEA) was performed for miR expression in TCGA GBM data set. Twenty miRs with top ratios between expression averages of PN and MES glioma spheres displayed enrichment in clinical PN GBM. (**c**) Relative expression levels of miR-125b and miR-20b of seven PN and five or six MES glioma spheres as indicated were determined with quantitative RT–PCR (qRT–PCR) assays. Error bars (s.d.) represent the data of triplicate samples for each glioma sphere line. **P<0.01, paired two-way Student's *t*-test. Data are representative from three independent experiments with similar results.

CSCs have a distinct miR signature in various cancers^18^,^26^. Therefore, we compared miR expression profiles of 654 human miRs in 8 glioma spheres, 3 PN (84, 17 and 20), 2 MES (83 and 1123), 528, 718 and 816 (ref. 9). Heat maps and principal component analysis (PCA)showed miR expression profiles clustered PN 84, 17 and 20, and MES 83 and 1123 (Fig. 1a; Supplementary Fig. 1a,b). Next, we found that, while the majority of miRs were expressed at low levels in GBM spheres, there are 20 miRs that displayed a twofold or higher expression in PN compared with MES spheres (Supplementary Fig. 1b). Then, we interrogated miR expression profiles in TCGA database with these top 20 PN-enriched miRs to examine their expression pattern in clinical PN and MES GBM tumours. These top 20 PN-enriched miRs effectively distinguish clinical PN from MES GBMs, based on clustering analysis (Fig. 1b; Supplementary Fig. 1c).

### miR-125b and miR-20b modulate MES and PN phenotypes

To identify miRs that influence the biological properties of glioma spheres, we predicted targets of miRs using StarBase v2.0 (http://starbase.sysu.edu.cn/)^27^. We focused on miRs: (1) that have at least a relative two-fold increase in PN spheres; (2) that target MES-associated genes *ALDH1A3*, *CD44*, and *HMGA2* (ref. 9), as well as components of the Notch, Hedgehog or Wnt pathways that are critical in CSCs^20^,^28^; and (3) that these miR-targeted genes were predicted by at least three databases within StarBase v2.0. Two miRs, miR-125b and miR-20b, met all these criteria. Thus, we examined the expression of miR-125b and miR-20b in our 14 glioma sphere panel from different sources: PN 17, 19, 157 and 84 (ref. 9); 11, 23, 34 (ref. 29); MES 30, 83 and 1123 (ref. 9); 20 and 28 (ref. 29); and 267 and 302 (ref. 8). As shown in Fig. 1c, levels of miR-125b and miR-20b were increased up to 7-fold and 40-fold, respectively, as measured by quantitative real-time PCR (qRT–PCR), in PN compared with MES spheres.

**Figure 2:**
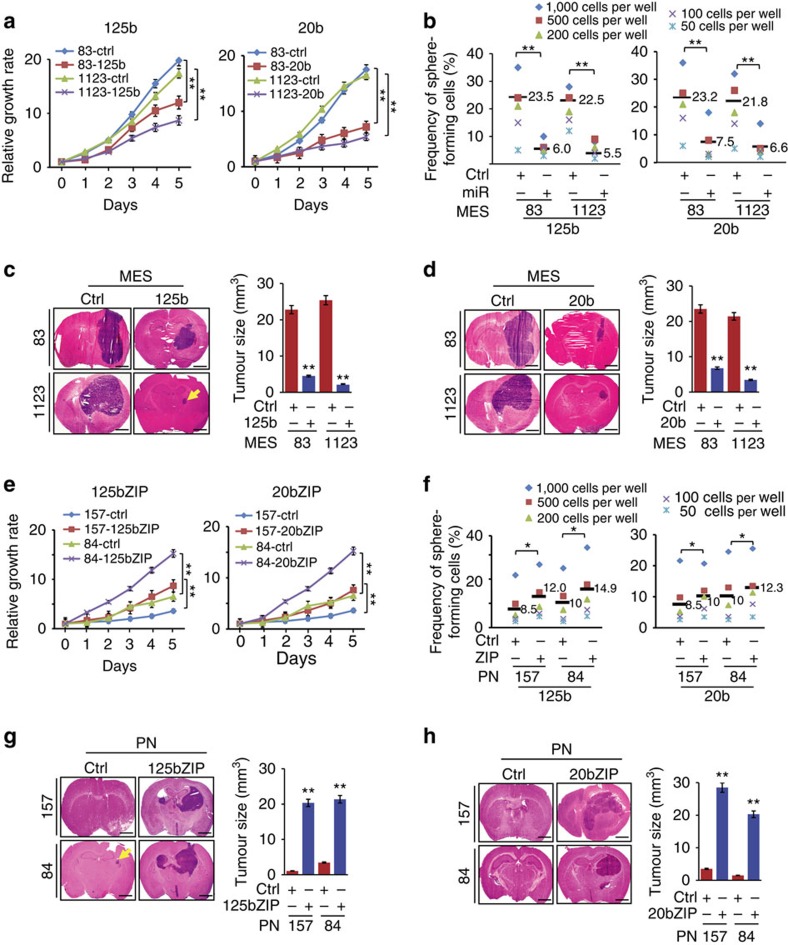
miR-125b and miR-20b regulate cell growth and tumorigenicity of PN and MES glioma spheres. (**a**–**d**) Ectopic expression of miR-125b and miR-20b, but not a control miR in MES 83 and 1123 glioma spheres inhibited cell proliferation (**a**). Sphere-forming ability (**b**) and tumorigenicity in brain xenografts in mice (*n*=5) that received modified MES 83 and 1123 (**c**,**d**) glioma spheres. Ctrl, a control miR. (**e**,**f**) Suppression of miR-125b and miR-20b by specific or a control miRZIP (sponges) in PN 84 and 157 glioma spheres promoted the cell proliferation (**e**). Sphere-forming ability (**f**) *in vitro*, and tumorigenicity in tumour xenografts (*n*=5) that received various PN 157 or 84 (**g**,**h**) glioma spheres. *In vitro* cell proliferation assays were performed in 12-well plates with 10,000 disassociated glioma sphere cells per well and counted at indicated times. *In vitro* neural sphere assays were performed with indicated cell numbers per well as described in the Methods section. Bar graphs in *in vivo* brain tumorigenicity assays are estimations of volumes of brain tumours formed by indicated glioma spheres. Scale bar, 1.0 mm. Error bars (s.d.) represent the data of triplicate samples for each glioma sphere line or brain sections with tumours from five mice per group. *P<0.05, **P<0.01, paired two-way Student's *t*-test. Data are representative from three independent experiments with similar results.

To determine the roles of these miRs in GBM, we first separately overexpressed miR-125b and miR-20b in MES 83 and 1123 spheres that have low endogenous expression of these miRs (Supplementary Fig. 2a). Phenotypically, overexpressed miR-125b and miR-20b markedly reduced cell growth rates and sphere formation of 83 and 1123 spheres (Fig. 2a,b). This was accompanied by the decreased expression of MES-associated genes, *ALDH1A3*, *CD44* and *HMGA2* (Supplementary Fig. 2b), minimal changes in levels of PN-associated genes, *SOX2* and *OLIG2*, and variable increases in Wnt signalling-related genes, *AXIN2* and *c-MYC* (Supplementary Fig. 2c). Next, we stereotactically implanted MES 83 and 1123 glioma spheres that stably express miR-125b, miR-20b or a control vector into the brains of mice. We found a significant reduction of GBM-like tumours derived from glioma spheres overexpressing miR-125b or miR-20b, but not the control vector (Fig. 2c,d). Consistent with this, overexpressed miR-125b and miR-20b inhibited MES-associated CD44 expression and had no effect on the levels of PN-associated SOX2 in these orthotopic brain tumour xenografts (Supplementary Fig. 2d,e).

Conversely, we individually antagonized expression of miR-125b and miR-20b in PN spheres using miRZIPs. Interestingly, cellular morphology of miR-125b or miR-20b miRZIP-inhibited PN spheres was transformed to one with MES-like irregular-shaped cell aggregates^9^. In sharp contrast, control PN spheres still displayed round sphere-like spheroids of tightly clustered cells (Supplementary Fig. 3a). Consistent with this phenotypic switch, inhibition of miR-125b or miR-20b increased the levels of MES-associated genes *ALDH1A3* and *CD44*, but reduced the levels of PN markers *SOX2* and *OLIG2* (ref. 9), and Wnt signalling-related genes *AXIN2* and *c-MYC* in PN 157 and 84 spheres (Supplementary Fig. 3b–d). Accompanying with these changes, PN 157 and 84 spheres, with inhibited miR-125b or miR-20b showed a substantial increase in their *in vitro* cell growth and sphere formation compared with controls (Fig. 2e,f). In animals, 15 days post implantation control PN 84 and 157 spheres do not form detectable tumour xenografts in the brain. In contrast, miRZIP-mediated inhibition of either miR-125b or miR-20b in PN 84 or 157 spheres resulted in significantly enhanced tumorigenicity in the brain (Fig. 2g,h). Furthermore, inhibition of miR-125b or miR-20b induced MES-associated CD44 expression, but did not alter the levels of PN-associated *SOX2* in these tumours (Supplementary Fig. 3e,f).

### Wnt signalling induces miR-125b and miR-20b in PN GBM

**Figure 3:**
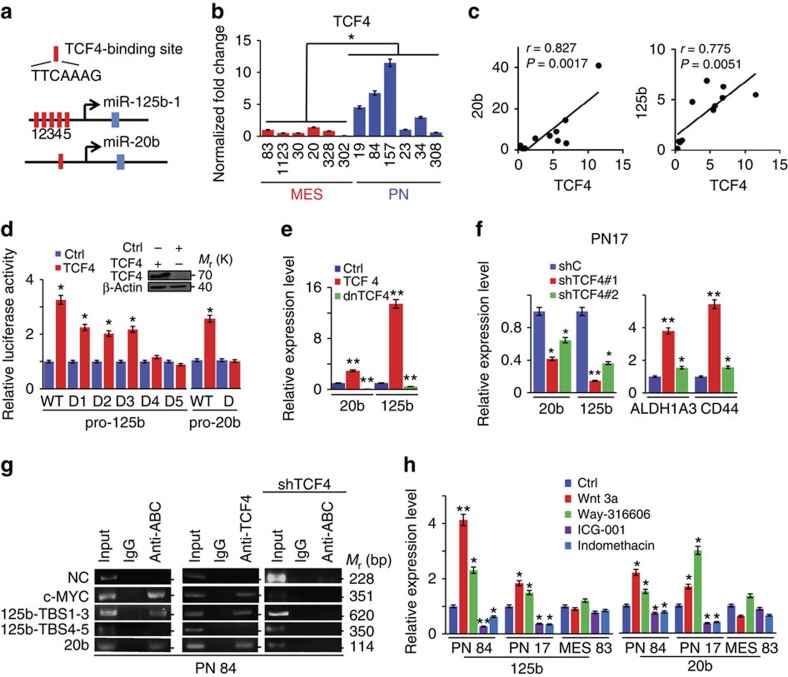
TCF4 induces expressions of miR-125b and miR-20b. (**a**) Schematic of each TCF4-binding site (TBS) in promoter regions of miR-125b-1 and miR-20b. Blue bar: coding region for miR. Arrows: transcription start sites. (**b**,**e**–**h**) qRT–PCR assays. (**b**) TCF4 is expressed at higher levels in PN spheres compared with MES spheres. (**c**) Correlation plots and statistics for expressions of TCF4 and miR-125b or miR-20b in six PN and six MES spheres. (**d**) Dual luciferase reporter assays. The miR-125b promoter with TBS 1–3 and the miR-20b promoter with a single TBS site are critical for TCF4 induction of these miRs, respectively. D, deletion. (**e**) WT TCF4 induces, while a dominant negative (dn) TCF mutant suppresses expression of miR-125b and miR-20b in 293T cells. Ctrl, a control vector. (**f**) Knockdown of TCF4 by two separate shRNAs inhibits expressions of miR-125b and miR-20b, but increased MES-associated genes *ALDH1A3* and *CD44* in PN 17 spheres. shC, a scramble shRNA control. (**g**) Chromatin immunoprecipitation (ChIP) assays using a control IgG, anti-active β-catenin (ABC), or anti-TCF4 antibodies in PN 84 spheres with or without knockdown of TCF4. A *c-MYC* gene promoter containing TBSs was used as a positive control. The coding region of β-actin was used as a negative control (NC). (**h**) Relative expressions of miR-20b and miR-125b in PN 84 and 17, and MES 83 spheres that were separately treated with Wnt activators Wnt3a (200 ng ml^−1^) or WAY-316606 (20 μM), and Wnt inhibitors ICG-001 (ICG; 5 μM), indomethacin (Indo; 5 μM), or a vehicle control for 24 h. In these qRT–PCR assays, a U6 small nuclear RNA (snRNA) was used as an internal control. Data were normalized to non-treated controls. Error bars (s.d.) represent the data of triplicate samples for each cell line. *P<0.05, **P<0.01, paired two-way Student's *t*-test. Data are representative from three independent experiments with similar results.

To determine the mechanisms that cause elevated levels of miR-125b and miR-20b in PN spheres, we used ALGGEN-PROMO^30^ to identify potential transcription factor-binding sites in the miR-125b and miR-20b promoter regions. In humans, mature miR-125b can be generated from two different precursor miRs (pre-miR-125b-1 on chromosome 11 and pre-miR-125b-2 on chromosome 21). We determined the expression of pre-miR-125b-1 and pre-miR-125b-2 by qRT–PCR. We found that pre-miR-125b-2 was detectable, but at much lower levels compared with pre-miR-125b-1 in PN spheres, whereas both precursors were at low levels in MES spheres (Supplementary Fig. 4a). Thus, we focused on the promoter of pre-miR-125b-1. We identified five putative TCF4-binding motifs within the miR-125b-1 promoter^31^ and one conserved TCF4-binding motif in the miR-20b promoter (Fig. 3a). Consistent with a prior study that TCF4/β-catenin contributes to GBM development^32^, TCF4 had higher expression in PN compared with MES spheres (Fig. 3b), and displayed a positive correlation with the expression of miR-125b and miR-20b in 12 PN and MES spheres (Fig. 3c). Next, we separately co-expressed wild-type (WT) promoters of miR-125b-1 and miR-20b, and their mutated counterparts together with an internal control vector in 293T cells with or without TCF4. Forced-expression of TCF4 induced activities of WT, but not mutated promoters of both miR-125b and miR-20b compared with the controls (Fig. 3d). Compared with the full-length promoters of miR-125b or miR-20b, deletion of TCF4-binding site(s) in miR-125b and miR-20b promoters completely (D4 or D5 of miR-125b and D of miR-20b) or moderately (D1, D2 and D3 of miR-125b) reduced their promoter activities (Fig. 3d). Conversely, WT TCF4 induced, and a dominant negative (dn) TCF4 suppressed, endogenous levels of miR-125b and miR-20b in these cells (Fig. 3e). Afterwards, we knocked down endogenous TCF4 in PN 17 spheres using two different short hairpin RNAs (shRNAs, Supplementary Fig. 4b). Inhibition of TCF4 resulted in a significant decrease in levels of miR-125b and miR-20b, and an increase in MES-associated *ALDH1A3* and *CD44* (Fig. 3f). Chromatin immunoprecipitation (ChIP) assays further confirmed chromatin occupancy of an activated β-catenin (ABC) and TCF4 at the promoter regions of miR-125b and miR-20b, respectively (Fig. 3g). Finally, when three glioma spheres were treated with Wnt stimulators Wnt3a or WAY-316606 (ref. 33), PN 84 and 17, but not MES 83, showed significant increases in the expression of miR-125b and miR-20b (Fig. 3h). In contrast, treatments of the spheres with Wnt Inhibitors ICG-001 or indomethacin^25^ resulted in decreases in miR-125b and miR-20b expression in PN, but not in MES spheres (Fig. 3h).

### Wnt signalling is activated in PN but not in MES GBM

**Figure 4:**
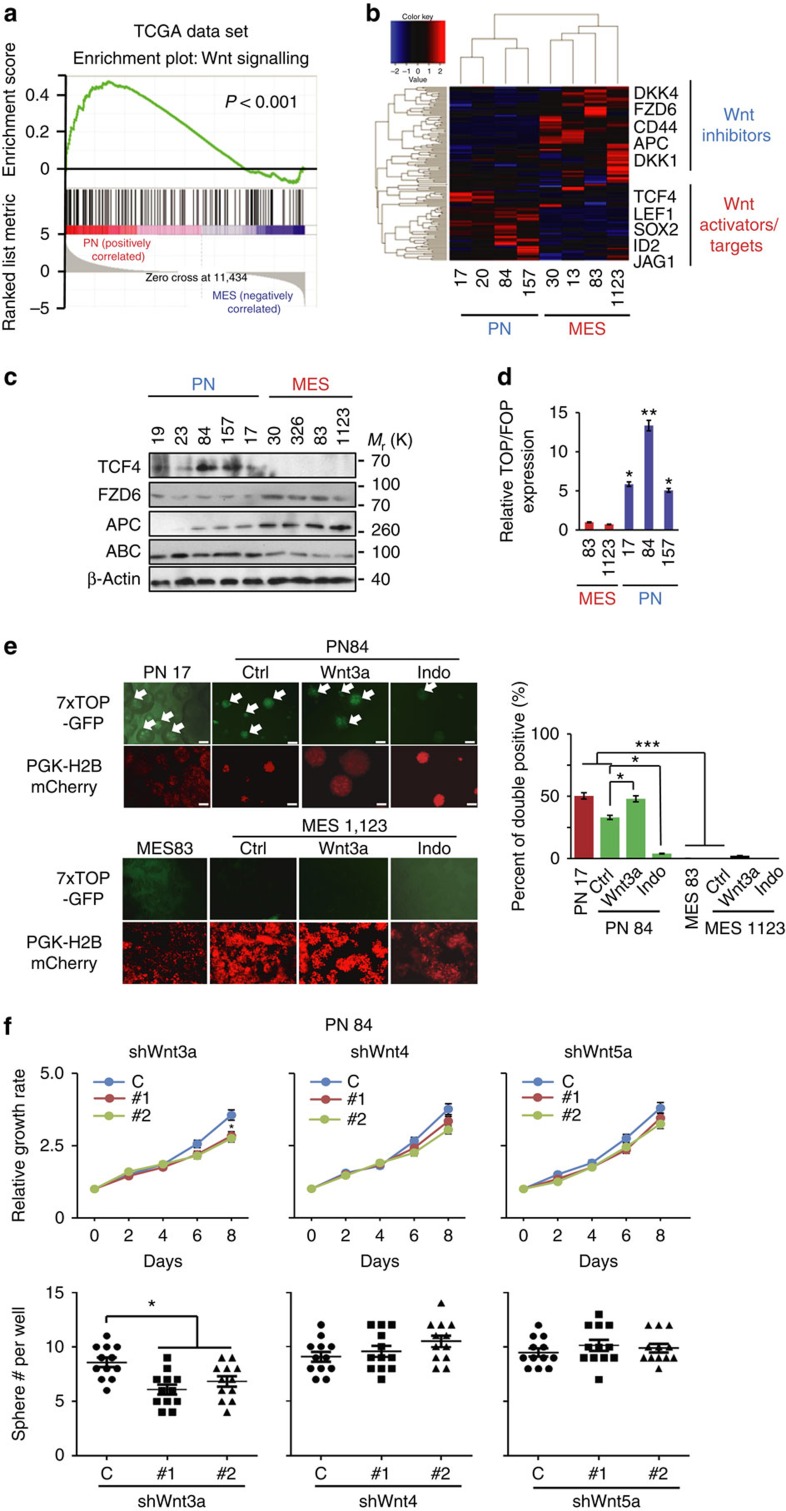
Wnt signalling pathway is active in PN but not in MES GBM. (**a**) Gene Set Enrichment Analysis (GSEA) of TCGA data set was performed for the expression of gene components of the canonical Wnt pathway in clinical PN and MES GBMs. Wnt signalling pathway is active in PN GBMs, but not in MES GBMs. (**b**) Hierarchical cluster analysis on expression data of the Wnt pathway genes differentially expressed between PN and MES spheres. (**c**) IB analysis of Wnt pathway components including TCF4, FZD6, APC, and activated β-catenin (ABC) in indicated PN and MES spheres. (**d**) TopFlash/FopFlash assays that quantify relative Wnt signalling activity in indicated PN and MES spheres. Values in bar graphs were normalized to those in MES 83. (**e**) Indicated PN and MES spheres were shown expressing GFP under the control of a 7 × TCF/LEF optimal promoter cassette (7 × TOP) and constitutively expressed nuclear mCherry. PN 84 and MES 1123 were treated with Wnt3a (200 ng ml^−1^), indomethacin (Indo; 20 μM), or a control (vehicle). Scale bar, 100 μm. Bar graph, quantification of fluorescent signal of GFP (TOP-GFP) versus mCherry (PGK-H2BmCherry). The percentage of GFP- and mCherry-positive spheres was determined by FACS. (**f**) Cell proliferation (upper panels) and sphere formation (lower panels) assays for PN 84 expressing control or specific shRNAs of *Wnt3a*, *Wnt4*, and *Wnt5a*, respectively. Error bars (s.d.) represent the data of triplicate samples for each cell line. *P<0.05, **P<0.01, paired two-way Student's *t*-test. Data in **a**–**f** are representative from three independent experiments with similar results.

To investigate the mechanism of regulation of miRs and Wnt signalling in GBM, we performed Gene Set Enrichment Analyses (GSEA) for the canonical Wnt pathway genes defined by the Kyoto Encyclopedia of Genes and Genomes (KEGG) on PN and MES GBM using TCGA data^3^ and our data set (GEO# GSE67089)^9^. We found that components of the Wnt signalling are enriched among differentially expressed genes when comparing PN with MES GBM (Fig. 4a; Supplementary Fig. 5a). Gene expression of the Wnt pathway segregates TCGA GBM samples into two groups, enriched in PN or MES subtypes (Supplementary Fig. 5b). Kaplan–Meier survival analysis of these two GBM subgroups based on Wnt gene expression predicted distinct survival outcomes (Supplementary Fig. 5c). Hierarchical clustering analysis also revealed differential expression of Wnt pathway genes in PN and MES spheres. High levels of positive regulators (*TCF4* and *LEF1*) and signature Wnt target genes (*SOX2*, *ID2* and *JAG1*) were found in PN spheres, whereas negative Wnt regulators, including *FZD6* and *APC*, were preferentially expressed in MES spheres (Fig. 4b). Differential expressions of *LEF1*, *FZD6*, *APC*, *SOX2*, *ID2* and *JAG1* were found in PN versus MES spheres (Supplementary Fig. 5d,e). TCF4 and ABC were also readily detected in PN spheres, but expressed at low levels or undetectable in MES spheres (Fig. 4c). Compared with PN spheres, MES spheres expressed Wnt inhibitors FZD6 and APC at higher levels (Fig. 4b,c; Supplementary Fig. 5d). TCF4 promoter activity was also higher in PN than MES spheres (Fig. 4d). Next, we measured TCF4 promoter activity in PN 17 and 84, and MES 83 and 1123 spheres using a lentiviral reporter (7 × TOP-GFP/PGK-H2BmCherry) with and without treatment of Wnt3a (activator) or indomethacin (inhibitor). We found that ∼35–50% of PN spheres were positive for eGFP and mCherry, and responded to Wnt3a stimulation or indomethacin inhibition. In contrast, mCherry-positive MES spheres displayed no detectable GFP signals and were unresponsive to treatment (Fig. 4e).

Among the 19 characterized Wnt ligands in humans, Wnt3a is the typified activator of canonical signalling, while Wnt5a is a potent inducer of non-canonical Wnt activity^20^,^21^,^22^. FZD1 mediates, while FZD6 inhibits, canonical Wnt signalling. FZD functions are also facilitated by several co-receptors^21^. Thus, we examined expression levels of the 19 Wnt ligands, 10 FZDs and co-receptors, LRP6, receptor tyrosine kinase-like orphan receptor 2 (ROR2), and receptor-like tyrosine kinase (RYK) in glioma spheres. In our gene expression data set (GEO# GSE67089)^9^, we found that all 19 Wnt ligands and most of 10 FZDs were expressed at low or moderate levels in PN and MES spheres. However, FZD3 and LRP6 were expressed at relatively higher levels in PN spheres, while FZD6 was higher in MES spheres (Supplementary Fig. 5f). Low levels of most Wnt ligands, but relatively higher levels of Wnt3a, Wnt4 and Wnt5a were found in four PN and two MES spheres (Supplementary Fig. 5g). Among the receptors, FZD6 expression was high in two MES, and FZD1, FZD2 and LRP6 were high in two PN, whereas other receptors displayed a mixed pattern of their expression in PN and MES spheres (Supplementary Fig. 5h). Finally, we knocked down Wnt3a, Wnt4 and Wnt5a using two separate shRNAs for each ligand in PN 84 and 157 spheres (Supplementary Fig. 5i). Inhibition of Wnt3a, but not Wnt4 or Wnt5a, attenuated cell growth and neural sphere formation in these PN glioma spheres (Fig. 4f; Supplementary Fig. 5j).

### miRs augment Wnt signalling through suppressing FDZ6

**Figure 5:**
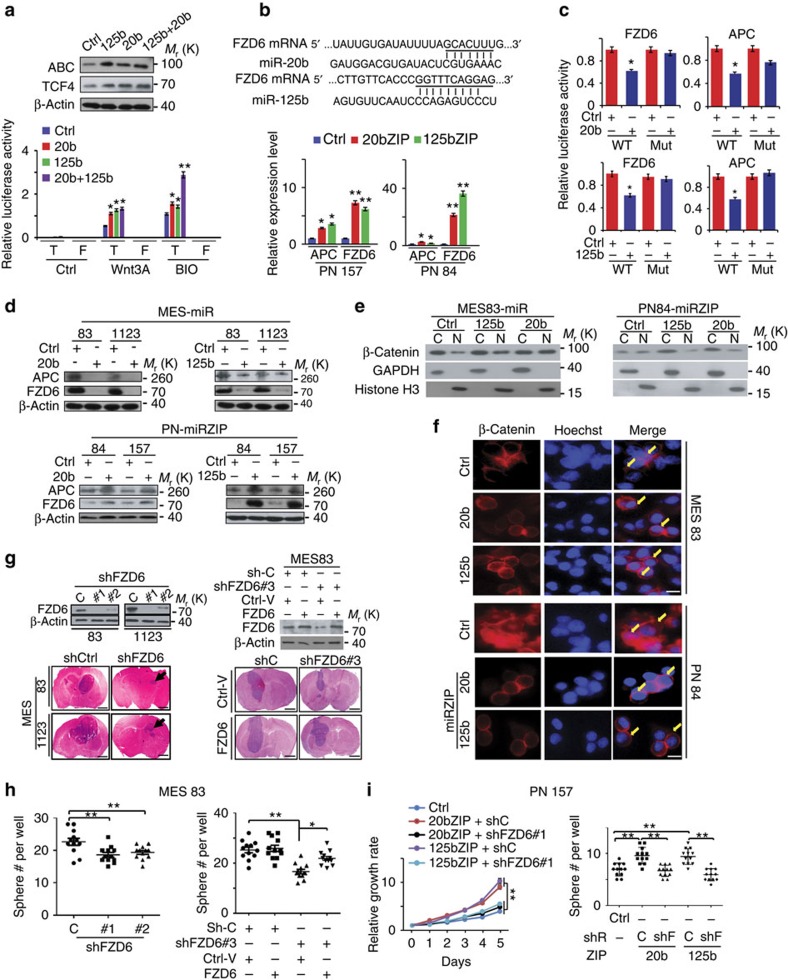
miR-20b and miR-125b augment Wnt signalling through targeting FZD6. (**a**) Top panel: IB of activated β-catenin (ABC) and TCF4 in miR-transduced 293T cells. Lower: cells were then treated with PBS (control), Wnt3a (200 ng ml^−1^) and BIO (2 μM), respectively. Firefly luciferase activities were normalized to Renilla luciferase activities. (**b**) Top: targeting sites of miR-20b and miR-125b (underlined, seed sequence) in mRNA of *FZD6* gene. Lower: qRT–PCR for expression of *FZD6* and *APC* in PN 157 (left) and PN 84 (right) expressing miR-20b ZIP, miR-125 ZIP or a non-targeting control miRZIP. β-Actin, an internal control. (**c**) Effects of miR-20b or miR-125b on the activities of indicated reporter genes containing WT or mutations at the miR-binding sites. Luciferase activities were normalized as in **a**. (**d**) IB for FZD6 and APC in miR-transduced MES 83 and 1123, or miRZIP-transduced PN 157 and 84. (**e**) Levels of β-catenin in cytoplasm (C) and cell nucleus (N) were examined in miR-transduced MES 83 or miR ZIP-transduced PN 84. GAPDH, control for cytoplasmic protein; Histone H3, control for nuclear protein. (**f**) Subcellular localization of β-catenin in glioma spheres. Yellow arrows, stained β-catenin. Scale bar, 20 mm. (**g**) Knockdown of FZD6 by specific shRNAs (#1/#2 targeting ORF of FZD6 mRNA and shRNA #3 targeting 3′-untranslated region (UTR) of FZD6 mRNA) inhibited growth of MES 83 and 1123 brain tumour xenografts. Re-expression of FZD6, but not a vector control, rescues shFZD6 #3-inhibited MES 83 tumour growth (*n*=5). Top panels: IB. Tumour areas in both experiments were quantified (Supplementary Fig. 6e). Scale bar, 1.0 mm. (**h**) sphere formation of MES 83 expressing control shRNA or shRNAs of FZD6, with or without re-expression of FZD6, or a control (right). (**i**) Cell proliferation (left) and sphere formation (right) for PN 157 expressing miR-20b ZIP, miR-125b ZIP, or a control with or without FZD6 knockdown. shR, shRNAs; C, control shRNA; shF, shRNA#1 for FZD6. Error bars (s.d.) represent the data of triplicate samples for each cell line or tumours from five individual mice in each group. *P<0.05, **P<0.01, paired two-way Student's *t*-test. Data are representative from three independent experiments with similar results.

On the basis of the above observations, we determined the effects of miR-125b and miR-20b on Wnt activity. Overexpression of miR-125b and miR-20b elevated levels of activated β-catenin (ABC) (non-phosphorylated) and TCF4 proteins in 293T cells. Furthermore, these miRs enhanced Wnt activation by Wnt3a or the GSK3β inhibitor BIO, measured by the ratio between luciferase activities of TOPFlash compared with the FOPFlash (Fig. 5a).

Next, we integrated miR target prediction using the program StarBase v2.0 (ref. 27) and analysis of gene profiling that are differentially expressed in PN versus MES spheres^9^. We identified two Wnt inhibitors FZD6 and APC that are expressed at low levels by PN compared with MES spheres (Fig. 4b) as potential targets for miR-125b^25^ and miR-20b (Fig. 5b, upper panel; Supplementary Fig. 7a). This prediction was validated by the data showing that miRZIP inhibition of miR-125b or miR-20b increased the expression of *APC* and *FZD6* in PN spheres (Fig. 5b, lower panel), whereas miR-125b or miR-20b suppressed activity of the *FDZ6* and *APC* gene reporters containing WT, but not mutated binding sites of miR-125b or miR-20b in 293T cells (Fig. 5c). Consistent with this, the expression of exogenous miR-125b or miR-20b in MES 83 and 1123 with low levels of these miRs, or separate inhibition of these miRs by miRZIPs in PN 157 and 84 with high levels of these two miRs, regulates the expression of FDZ6 and APC proteins (Fig. 5d).

We further determined the effects of miR-125b or miR-20b on subcellular localization of β-catenin^21^. Overexpression of miR-125b or miR-20b in MES spheres increased the amount of β-catenin in the cell nucleus, whereas inhibition of miR-125b or miR-20b by miRZIPs in PN spheres increased cytoplasmic β-catenin compared with controls (Fig. 5e,f; Supplementary Fig. 6a). Knockdown of endogenous FZD6 in MES 83 and 1123 (Fig. 5g) significantly decreased sphere formation and cell proliferation of MES spheres *in vitro*, (Fig. 5h; Supplementary Fig. 6b,c) and tumorigenicity of MES 83 and 1123 spheres in brain xenografts (Fig. 5g; Supplementary Fig. 6d,e). Consistently, inhibition of FZD6 also markedly reduced MES-associated CD44 expression in MES brain xenograft tumours (Supplementary Fig. 6f). In contrast, in PN 157 and 84, with high endogenous miR-125b and miR-20b and low FZD6, suppression of either miR by miRZIPs augmented cell growth and sphere formation, whereas knockdown of FZD6 rescued the capacity of these PN spheres to proliferate and form spheres (Fig. 5i; Supplementary Fig. 6g). Re-expression of shRNA-resistant FZD6 (Fig. 5g, right upper panel) in MES spheres rescued their ability for cell growth and sphere formation *in vitro*, and restored brain tumour xenograft growth that was reduced by FZD6 knockdown (Fig. 5g,h; Supplementary Fig. 6b–e).

Finally, we examined whether APC also plays a role in the growth and signalling of glioma spheres. Knockdown of APC by shRNAs had minimal impact on the cell growth and sphere formation of MES spheres (Supplementary Fig. 7b,c). Moreover, in PN 157 and 84, inhibition of APC reversed decreased ABC, but failed to prevent the loss of TCF4 caused by miRZIP inhibition of miR-125b or miR-20b in PN spheres (Supplementary Fig. 7d). Knockdown of APC with concomitant inhibition of miR-125b or miR-20b by miRZIPs also did not affect their cell proliferation and sphere formation (Supplementary Fig. 7e,f).

### FZD6 inhibits Wnt signalling through TAK1 in GBM

**Figure 6 f6:**
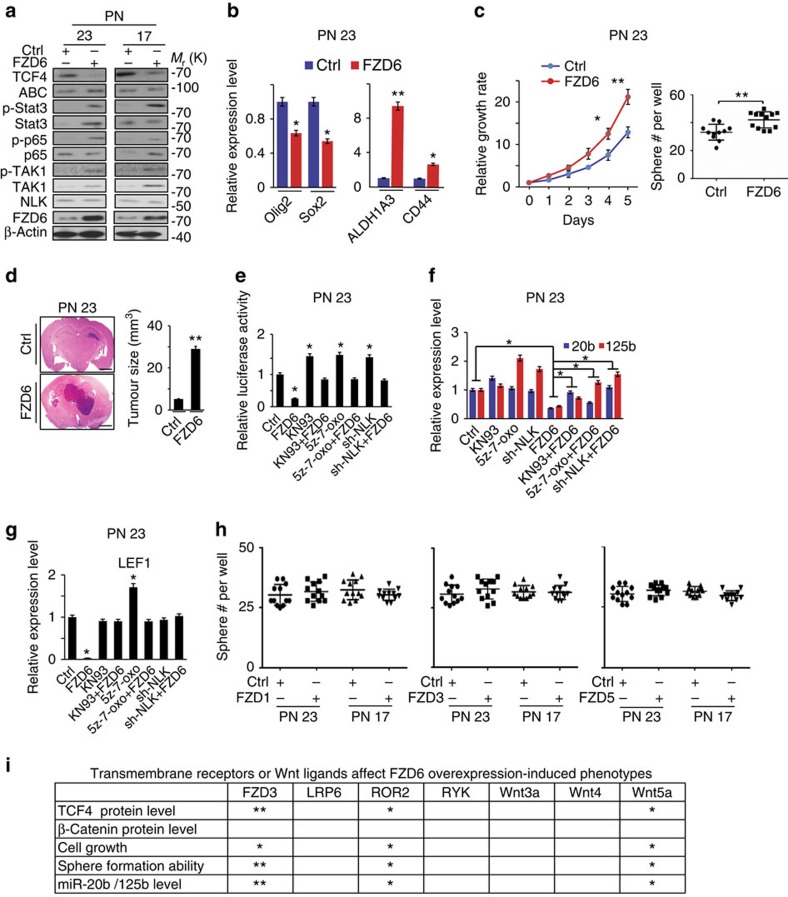
FZD6 mediates induction of the MES-associated phenotype and suppression of the Wnt signalling through TAK1. (**a**) IB analysis on PN 23 and 17 spheres with or without FZD6 overexpression using indicated antibodies. (**b**) qRT–PCR analysis of PN-associated genes *OLIG2* and *SOX2*, and MES-associated genes *ALDH1A3* and *CD44* in PN 23 spheres with or without FZD6 overexpression. (**c**) FZD6 expression promoted PN 23 spheres proliferation and self-renewal. (**d**) Ectopic expression of FZD6 promoted tumour growth of PN 23 spheres in GBM xenografts in the brain (*n*=5). Scale bar, 1.0 mm. (**e**) TOPFlash/FOPFlash assays on PN 23 with overexpressing FZD6 or control, with or without CaMKII inhibitor KN93 (5 μM), TAK1 inhibitor 5Z-7-oxo (5 μM), or a shRNA-NLK. (**f**,**g**) qRT–PCR analyses of the relative expression of miR-20b and miR-125b (**f**), and Wnt signalling target gene *LEF1* (**g**) in PN 23 overexpressing FZD6, or a vector control, with or without CaMKII inhibitor KN93 (5 μM), TAK1 inhibitor 5Z-7-oxo (5 μM), or a shRNA-NLK. (**h**) sphere formation assays for PN 23 and 17 spheres with or without overexpression of FZD1, FZD3, and FZD5, respectively. (**i**) Summary of data shown in Supplementary Fig. 10. An overview of transmembrane receptors and Wnt ligands that affect FZD6 overexpression-induced phenotypes. Data were normalized to the control. *Relative inhibitory effects on indicated protein expression or cellular properties. Error bars (s.d.) represent the data of triplicate samples for each cell line or tumours from five individual mice in each group. *P<0.05, **P<0.01, paired two-way Student's *t*-test. Data are representative from three independent experiments with similar results.

FZD6 is a member of the FZD family and a negative regulator of Wnt signalling through a calmodulin-dependent protein kinase II (CaMKII)–transforming growth factor-β-activated kinase 1 (TAK1)–NEMO-like kinase (NLK) pathway (CaMKII–TAK1–NLK)^34^. We thus assessed whether FZD6 regulates Wnt signalling and tumorigenicity in glioma spheres. Overexpression of FZD6 in PN 23 and 17 reduced the level of TCF4, but had minimal effects on ABC (Fig. 6a). In PN 84 and 157, miRZIP inhibition of miR-125b or miR-20b reduced TCF4 (Supplementary Fig. 8a). Such suppression was partially rescued by shRNA knockdown of FZD6 in PN spheres. Moreover, FZD6 inhibition could not rescue decreases in ABC caused by miRZIPs (Supplementary Fig. 8a). Interestingly, overexpression of FZD6, as well as miRZIP inhibition of miR-125b or miR-20b induced phosphorylation of the NF-κB p65 subunit, STAT3, and TAK1 in PN spheres (Fig. 6a and Supplementary Fig. 8a, respectively).

We then determined the effects of FZD6 expression on PN spheres. FZD6 overexpression resulted in decreases in PN markers *OLIG2* and *SOX2* and increases in MES-associated genes *ALDH1A3* and *CD44* in PN spheres (Fig. 6b; Supplementary Fig. 8b). Ectopic FZD6 expression in PN spheres increased cell proliferation and sphere formation *in vitro* (Fig. 6c; Supplementary Fig. 8c), enhanced brain tumorigenicity (Fig. 6d), upregulated expression of MES-associated CD44, while inhibiting PN-associated SOX2 *in vivo* (Supplementary Fig. 8d). Next, we examined the roles of the FZD6–CaMKII–TAK1–NLK pathway in PN spheres by small-molecule inhibitors for CaMKII, TAK1, or shRNA knockdown of NLK (Supplementary Fig. 8e). Overexpression of FZD6 in PN spheres reduced Wnt activity (Fig. 6e; Supplementary Fig. 8f), decreased expression of miR-125b and miR-20b (Fig. 6f; Supplementary Fig. 8g), and Wnt target genes *LEF1* and *ID2* (Fig. 6g; Supplementary Fig. 8h). Moreover, inhibition of CaMKII, TAK1, or knockdown of NLK blocked these FZD6-mediated effects (Fig. 6e–g; Supplementary Fig. 8f–h). Finally, we expressed exogenous FZD6 in MES spheres expressing miR-125b or miR-20b. FZD6 overexpression rescued miR-125b- or miR-20b-attenuated p-TAK1, p-p65, p-STAT3 (Supplementary Fig. 9a), cell growth (Supplementary Fig. 9b), and sphere formation (Supplementary Fig. 9c).

Among 10 FDZ family members and co-receptors, FZD1, FZD3, FZD5, and LRP6, RYK, and RORs are well-established receptors and co-receptors that mediate Wnt signalling, respectively^35^,^36^. FZD3, FZD5, and LRP6 were expressed at high levels in PN compared with MES spheres, while RYK and FZD8 were found in both PN and MES spheres (Supplementary Fig. 5f–h). However, separate overexpression of FZD1, FZD3, and FZD5 in PN 23 and 17 spheres had no effect on the expression of TCF4, cell growth, and sphere formation of PN spheres *in vitro* (Fig. 6h; Supplementary Fig. 10a,b). Moreover, when FZD3 or co-receptors LRP6, ROR2, and RYK were separately depleted by shRNAs in FZD6-overexpressing PN 23 or 17 spheres, inhibition of FZD3 and ROR2, but not LRP6 and RYK, attenuated FZD6-regulated TCF4 expression, phosphorylation of p65, STAT3, and TAK1 (Fig. 6i; Supplementary Fig. 10c), sphere formation, and proliferation of PN spheres (Supplementary Fig. 10e,f). They also rescued the suppressed levels of miR-20b and miR-125b caused by FZD6 overexpression (Fig. 6i; Supplementary Fig. 10g). Finally, knockdown of ligand Wnt5a, but not Wnt3a or Wnt4, affected FZD6-overexpressing spheres, altering Wnt and cellular signalling, cell growth, and sphere formation of PN spheres (Figs 4f or 6i, [Fig f6]; Supplementary Fig. 10c–g).

### Targeting activated signalling pathways inhibits GBM subtypes

**Figure 7:**
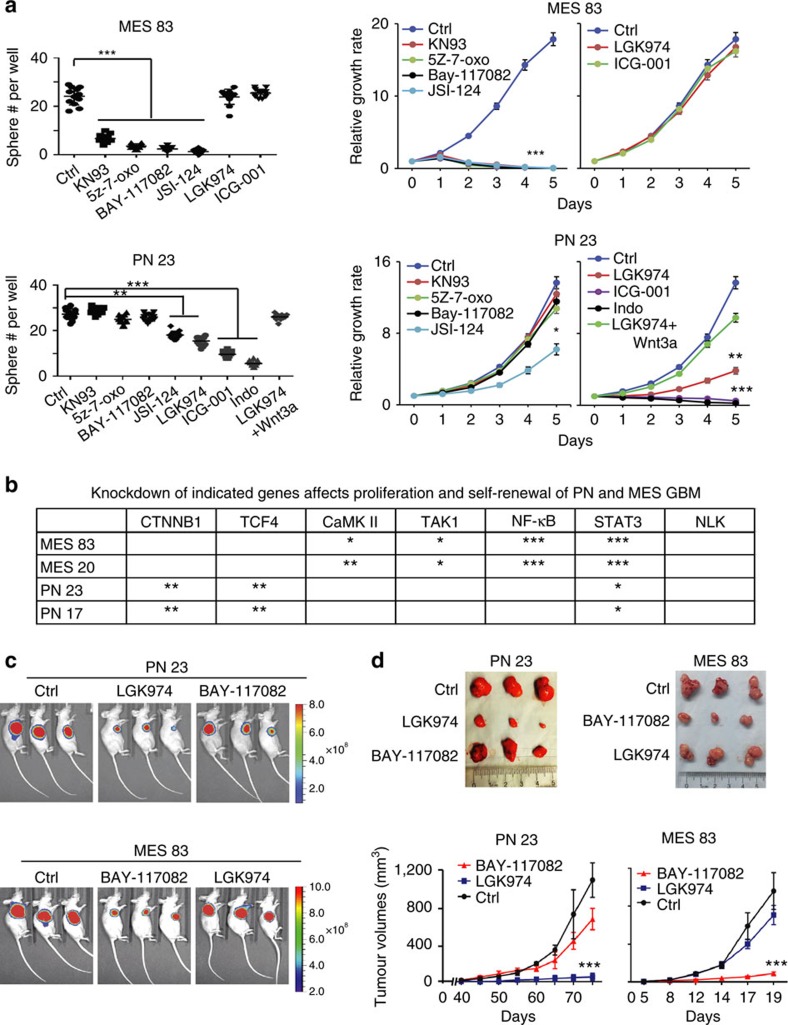
PN and MES GBM subtypes can be selectively inhibited with pathway-specific inhibitors. (**a**) Sphere formation and cell proliferation of indicated glioma spheres treated with DMSO (Ctrl), KN93 (5 μM), 5Z-7-oxo (5 μM), BAY-117082 (5 μM), JSI-124 (0.1 μM), LGK974 (5 μM), ICG-001 (5 μM), or LGK974 (5 μM)+Wnt3a (200 ng ml^−1^), respectively. (**b**) Summary of data shown in Supplementary Fig. 11. An overview of knockdown of β-catenin (CTNNB1), TCF4, CaMK II, TAK1, NF-κB, STAT3, or NLK that reduce proliferation and self-renewal abilities of PN and MES spheres. Data were normalized to the control. *Relative inhibitory effects on indicated protein expression or cellular properties. *P<0.05, **P<0.01, ***P<0.001. (**c**) Effects of Wnt inhibitor LGK974 (3 mg kg^−1^) and NF-κB inhibitor Bay-117082 (10 mg kg^−1^) on tumour xenografts established subcutaneously by PN 23 or MES 83 spheres. Indicated glioma spheres (1 × 10^6^ cells) were implanted into the flanks of nude mice. Treatments were started when palpable tumours appeared. Representative bioluminescent images (left) are shown. (**d**) Dissected flank tumours (upper) and tumour growth kinetics (lower) from different groups are shown. Error bars (s.d.) represent the data of triplicate samples for each cell line or tumours from five individual mice in each group. *P<0.05, **P<0.01, ***P<0.001, paired two-way Student's *t*-test. Data are representative from three independent experiments with similar results.

We tested whether two subtypes of glioma spheres respond differently to inhibitions that target GBM subtype-enriched signalling pathways. We treated two PN and two MES glioma spheres separately with inhibitors of Wnt signalling, CaMKII, TAK1, NF-κB, and STAT3. Inhibitors of CaMKII, TAK1, and NF-κB significantly decreased sphere formation and cell proliferation of MES but not PN spheres. Conversely, PN but not MES spheres were more sensitive to the Wnt inhibitor LGK974, which selectively targets porcupine, a Wnt-specific acyltransferase required for the processing of Wnt ligands^37^. In addition, Wnt activator Wnt3a rescued LGK974-suppressed sphere formation and cell growth of PN spheres. On the other hand, compared with PN spheres, MES spheres appeared more sensitive to the STAT3 inhibitor, JSI-124 (Fig. 7a; Supplementary Fig. 11a). Next, we knocked down the following signalling molecules in PN or MES spheres using two separate shRNAs for each gene and a scrambled control: CaMKII, TAK1, NF-κB, STAT3, TCF4, β-catenin and NLK (Supplementary Fig. 11b). In MES spheres, inhibition of CaMKII, TAK1, NF-κB and STAT3, but not β-catenin, and NLK, reduced their capabilities of cell growth and neural sphere formation. In contrast, in PN spheres, inhibition of TCF4, β-catenin, and STAT3 attenuated proliferation and sphere formation. However, suppression of CaMKII, TAK1, NF-κB, and NLK had no effect on growth (Fig. 7b; Supplementary Fig. 11c,d). Last, we treated mice bearing GBM xenografts established by PN or MES spheres with inhibitors of Wnt (LGK974), or NF-κB (BAY-117082)^38^ pathways. PN GBM xenografts responded well to LGK974, but only moderately to BAY-117082. Conversely, growth of MES GBM xenografts was markedly suppressed by BAY-117082, but not LGK974 (Fig. 7c,d). Inhibition of subtype-enriched signalling in PN (Wnt) or MES (NF-κB) tumours by LGK974 or BAY-117082, was also subtype-specific on their corresponding gene targets within PN or MES GBM tumour xenografts (Supplementary Fig. 12).

### TCF4/miR-125b/miR-20b/FZD6 pathway is clinically prognostic

**Figure 8:**
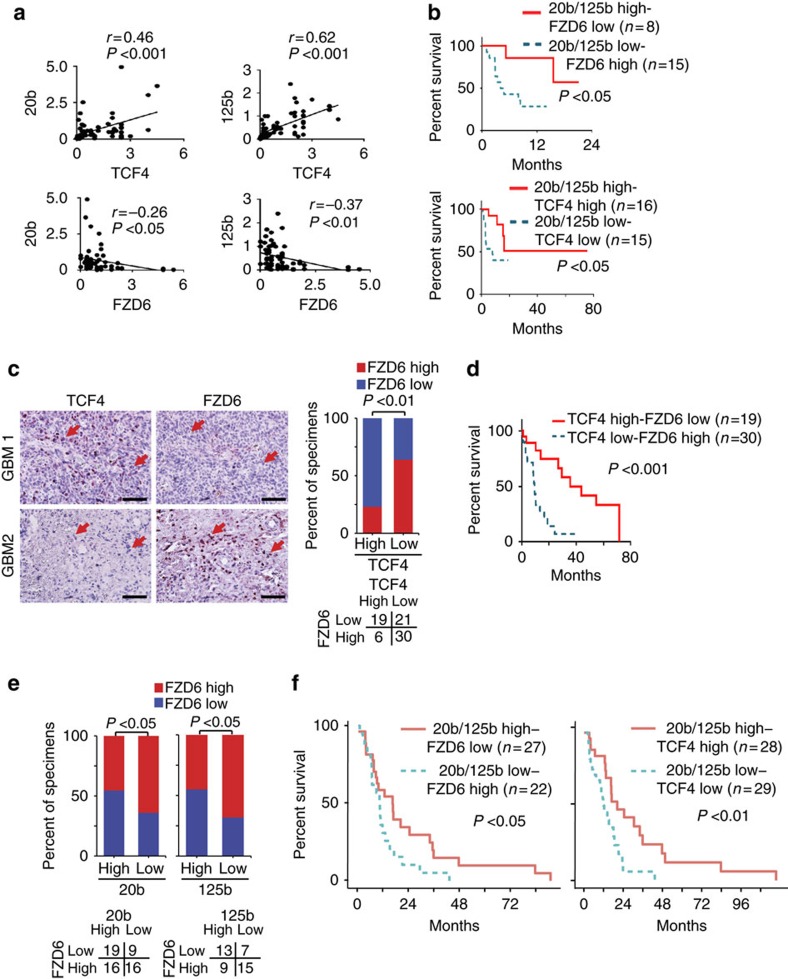
Correlative expressions of Wnt signalling modulators and miR-126 or miR-20b are prognostic for clinical GBM. (**a**) Correlation of expression between miR-20b or miR-125b, and TCF4 or FZD6 in 61 snap-frozen clinical GBM tumour specimens. (**b**) Kaplan–Meier curves for survival of patients with GBM that have expression levels of miR-20b/miR-125b high and FZD6 low (upper), miR-20b/miR-125b high and TCF4 high (lower). (**c**) Representative images of IHC of TCF4 and FZD6 in a separate cohort of a total of 76 paraffin-embedded clinical GBM samples. Arrows, corresponding tumour cells in sister tumour sections that were stained positive or negative by indicated antibodies. Right, bar graphs indicate percentages of specimens showing high or low TCF4 expression relative to level of FZD6. Scale bar, 50 μm. (**d**) Kaplan–Meier analyses for survival of patients with GBM that have expression levels of TCF4 high/FZD6 low or TCF4 low/FZD6 high. (**e**) Percentages of specimens showing high or low miR-20b or miR-125b expression relative to levels of FZD6 in TCGA data sets. (**f**) Kaplan–Meier curves for survival of patients with GBM that have expression levels of miR-20b/miR-125b high and FZD6 low (left), and miR-20b/miR-125b high and TCF4 high (right) in TCGA data sets. In **b**,**d** and **f**, *P* values were calculated using log-rank and Gehan–Breslow–Wilcoxon tests. *P<0.05, **P<0.01, ***P<0.001, paired two-way Student's *t*-test. Data are representative from three independent experiments with similar results.

Last, we determined the expression of miR-125b and miR-20b by qRT–PCR, and the expression of TCF4 and FZD6 by immunoblotting (IB) in 61 clinical snap-frozen GBM samples. There was a positive correlation between the expression of TCF4 and miR-125b or miR-20b, and an inverse correlation between FZD6 and miR-125b, or miR-20b (Fig. 8a; Supplementary Fig. 13a). Kaplan–Meier survival analysis, log-rank and the Gahan–Breslow–Wilcoxon tests revealed that low expression levels of miR-125b or miR-20b correlated with a relatively shorter patient survival (long-rank test, *P*<0.05; Supplementary Fig. 13b). Conversely, a worse prognosis is predicted in patients with low expression of either miRs combined with high expression of FZD6 or low expression of TCF4 (Supplementary Fig. 13c). Kaplan–Meier analyses that combined expression levels of miR-20b/miR-125b with FZD6 or TCF4 expression also demonstrates a statistically significant correlation with survival in these GBMs (Fig. 8b). Next, we performed immunohistochemical (IHC) staining for the expression of TCF4 and FZD6 in a separate cohort of 76 paraffin-embedded GBM tumours. GBM patients with higher levels of TCF4, lower levels of FZD6 or TCF4 high/FZD6 low had a longer overall survival, whereas GBM patients with lower TCF4 expression or higher FZD6 expression had worse prognosis (Fig. 8c,d; Supplementary Fig. 13d). Finally, in the TCGA data sets of clinical PN and MES GBM samples, low levels of miR-20b, but not miR-125b, was strongly correlated with improved survival outcomes (Supplementary Fig. 14a,b). FZD6 expression alone showed a trend towards shorter patient survival, but did not reach statistical significance (log-rank test, *P*=0.137), unless it was combined with miR-20b expression (Supplementary Fig. 14c,d). Elevated TCF4 expression alone, or in combination with miR-20b, is correlated with improved survival (Supplementary Fig. 15a,c). Expression levels of miR-20b and miR-125b positively correlated with TCF4, but inversely correlated with FZD6 (Fig. 8e; Supplementary Fig. 15e). Last, combining low levels of miR-20b/125b with high levels of FZD6 or low levels of TCF4 predicts shorter survival of patients with these GBMs (Fig. 8f). Taken together, these data strongly support the utilization of miR-125b, miR-20b, TCF4, and FZD6 levels in outcome predictions of clinical GBMs and may help guide treatment options.

## Discussion

**Figure 9:**
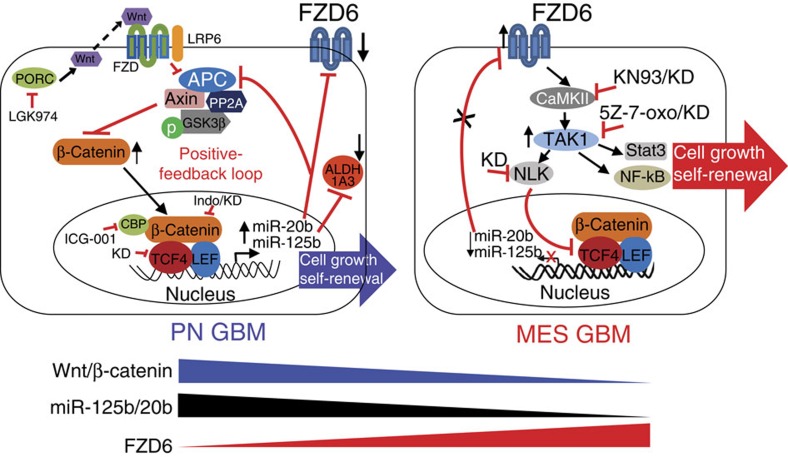
Model that summarizes the proposed regulatory circuit that controls GBM phenotypes. Wnt/β-catenin signalling is active in PN, but inactive in MES GBM, and upregulates expression of miR-125b and miR-20b that in turn repress APC and FZD6 to sustain Wnt/β-catenin signalling, thereby leading to cell growth and self-renewal of PN GSCs. FZD6 acts as a negative regulator of Wnt/β-catenin signalling by activating the CaMKII–TAK1–NLK pathway, which promotes STAT3 and NF-κB signalling that are critical for the MES-associated phenotype. Targeting differentially enriched signalling pathways in PN versus MES GBM effectively inhibits distinct GBM subtypes.

In this study, we describe a regulatory circuit composed of miR-125b/miR-20b and Wnt signalling that regulate PN and MES phenotypes of GBM through FZD6 (Fig. 9). In PN GBM, canonical Wnt signalling is highly active and induces transcription of miR-125b and miR-20b. miR-125b and miR-20b target Wnt inhibitors FZD6 and APC, further enhancing Wnt activity. In MES GBM, high levels of FDZ6 attenuate Wnt signalling through the CaMKII–TAK1–NLK pathway, decreasing the levels of miR-125b and miR-20b. Activated TAK1 in turn stimulates NF-κB and STAT3 pathways that control the MES-associated phenotype of GBM^29^,^39^. These two regulatory loops regulate distinct phenotypes of GBM (Fig. 9).

In this study, we report that miR profiles clearly distinguish PN from MES spheres. Interestingly, spheres 816 and 528 that displayed both PN- and MES-like properties^9^ are clustered between PN and MES spheres by miR profiling analysis. These data indicate that miR signatures can be used to classify human GBMs, similar to subtyping by their gene expression signatures^2^,^4^,^8^,^9^. Our results are consistent with a previous study showing that expression profiling of 121 miRs in 216 GBMs in TCGA database identified five distinct GBM subclasses^26^. Thus, miRs not only characterize genetically defined subtypes of human cancers, but also regulate malignant tumour phenotypes.

miR-125b and miR-20b displayed context-dependent properties in regulating tumorigenicity of various cancers^40^,^41^,^42^. Here we show that regulation of GBM tumorigenicity by miR-125b and miR-20b is subtype-dependent. In PN GBM, high levels of these two miRs suppress two Wnt pathway inhibitors, APC and FZD6, enhancing the Wnt signalling and inhibiting a MES-associated phenotype. Significantly, the Wnt activity is enriched in PN GBM compared with MES GBM. TCF4, a key transcriptional factor in Wnt pathway, induces transcription of miR-125b and miR-20b. Modulation of these miRs that target Wnt inhibitors FZD6 and APC affects nuclear localization of β-catenin in both PN and MES glioma spheres. We also report that in PN spheres, Wnt3a, but not Wnt4 and Wnt5a, is responsible for the activation of Wnt signalling. Enhanced Wnt activity in PN GBM is consistent with the oncogenic role of the Wnt pathway in GBM^32^. In MES GBM, miR-125b and miR-20b are suppressed, while the Wnt inhibitor FZD6 is highly expressed. The upregulated FZD6 exhibits dual roles. FZD6 inhibits the Wnt activity through the CaMKII–TAK1–NLK pathway, decreasing miR-125b and miR-20b expression. Alternatively, FZD6-activated TAK1 (ref. 34) stimulates NF-κB and STAT3 signalling that are essential in maintaining a MES phenotype in GBM^29^,^39^. These regulatory events were confirmed by the modulation of miR-125b, miR-20b, TCF4, FZD6, CaMKII, TAK1 and NLK through ectopic expression, miRZIPs, shRNA knockdown and targeted drug inhibitors. Such distinct regulation was further validated in clinical GBM tumours. Thus, our results establish a distinct regulatory circuit composed of miR-125b/miR-20b, the Wnt pathway, and MES-associated genes. This tumour subtype-dependent regulatory circuit is composed of distinct mediators. Thus, it is plausible that similar to previously described results^10^,^12^,^43^, in response to genetic mutations or therapeutic treatments, this regulatory circuit modulates GBM phenotypes.

FZD6 inhibits Wnt signalling at different nodes in the Wnt pathway^21^ and has been implicated in cancer development^44^,^45^. We show that FZD6 is highly expressed by MES spheres and directly suppressed by miR-125b and miR-20b. Inhibition of FZD6 attenuates tumorigenic behaviours of GBM *in vitro* and *in vivo*. Conversely, re-expression of FZD6 in MES spheres with shRNA-ablated endogenous expression reverted the phenotype. Conversely, ectopic expression of FZD6 in PN spheres inhibits PN-associated Wnt activity through the CaMKII–TAK1–NLK pathway. FZD6 also promotes tumorigenicity of PN GBM *in vivo* and induces a MES-like phenotype, while decreasing PN markers, *OLIG2* and *SOX2*. Concomitantly, high levels of FZD6 in glioma spheres promote the MES phenotype of GBM by stimulating MES-associated STAT3 and NF-κB pathways^29^. TAK1 is an important component of TGF-β signalling and regulates both NF-κB and MAPK signalling pathways^46^. TAK1 was found essential for EGFRvIII-mediated tumorigenicity in GBM through NF-κB activation^47^. We show that inhibition of TAK1 decreases self-renewal of MES but not PN spheres. Among Wnt ligands, receptors and co-receptors, we found that FZD3, ROR2, and Wnt5a regulate FZD6 overexpression-induced phenotypes in glioma spheres. Moreover, the effects of inhibition of either FZD3 or ROR2 on FZD6 were moderate, thereby corroborating with previous reports of functional redundancies of FZD6 with FZD3 (ref. 36) and ROR2 as an alternative receptor for Wnt5a^22^. Last, these findings were further confirmed by complimentary analyses of 61 snap-frozen GBM tumour tissues, a separate set of 76 paraffin-embedded GBM tumour samples and TCGA expression data. Expression of FZD6 inversely correlates with miR-125b/miR-20b and TCF4 levels, and combined expression levels of miR-20b/-125b, with either FZD6 or TCF4, effectively predict GBM patients' survival. Interestingly, our data revealed a reciprocal expression pattern of TCF4 and FZD6 in GBM subtypes. In PN GBM, TCF4 induces expression of miR-125b and miR-20b that in turn inhibit expression of FZD6 and several other targets. We currently do not know the precise mechanism by which high levels of FZD6 found in MES GBM suppress TCF4 expression. However, FZD6 inhibition of TCF4 would be consistent with findings that FZD6 antagonizes canonical Wnt signalling through the TAK1–NLK pathway^34^,^48^ and NLK-associated RING finger protein (NARF). NARF is required for NLK kinase activity and destabilizes TCF4 by inducing TCF4 ubiquitination^49^. Collectively, FZD6-activated TAK1 suppresses Wnt signalling through NLK, and enhancing STAT3 and NF-κB pathways, thereby maintaining a MES-associated phenotype in GBM.

Here we also show that the known Wnt inhibitor LGK974 (ref. 37), was more effective against PN GBM than the NF-κB inhibitor Bay-117082 (ref. 38). In contrast, Bay-117082 was more effective than LGK974 at inhibiting MES GBM tumorigenicity. Inhibition of subtype-specific pathways was demonstrated using either small-molecule inhibitors for these targets or gene knockdown studies of each key component in the Wnt signalling, NF-κB and STAT3 pathways. These data suggest that subtypes of GBM with distinctly enriched oncogenic signalling can be selectively treated with pathway-specific inhibitors. However, since we do not know the bioavailability of these two small inhibitors in the brains of our orthotopic GBM xenografts, further exploration of this approach using pathway-specific inhibitors with validated bioavailability data in combination with non-invasive imaging technologies are warranted.

In conclusion, this study identifies a regulatory circuit, composed of miR-125b, miR-20b, Wnt signalling and FZD6, that regulates PN and MES phenotypes of GBM. These results provide the underlying mechanisms that maintain GBM PN and MES properties, and potentially regulate PN to MES transition. We also demonstrate that targeting distinctly enriched oncogenic pathways can selectively suppress different GBM subtypes. Further validation of our findings will lead to the development of more effective personalized therapies for patients with malignant tumours.

## Methods

### Cell lines and cell culture

Human embryonic kidney 293T cells from American Type Culture Collection were maintained in DMEM supplemented with 10% fetal bovine serum, and 1% penicillin and streptomycin. Patient-derived glioma spheres, which were recently characterized and origins confirmed^8^,^9^,^29^, were cultured in DMEM/F12 (Invitrogen), supplemented with B27 (2%, Invitrogen), penicillin and streptomycin (1%, Invitrogen), heparin (5 μg ml^−1^, Sigma-Aldrich), epidermal growth factor (20 ng ml^−1^) and basic fibroblast growth factor (20 ng ml^−1^, Peprotech), and grown in suspension in plates or flasks with filter caps. Glioma spheres were expanded by changing half of the media approximately every 2 days. Glioma spheres were passaged by pelleting the cells with low-speed centrifugation (200*g* for 2 min). After removing supernatant, cells were dissociating the pellet using gentile mechanical up-and-down pipetting. If needed, enzymatic dissociation was employed with StemPro Accutase (1 ml, Invitrogen). Cell lines were cultured in water-jacketed humidity-controlled incubators at 37 °C and 5% CO_2_. Cell transfections or infections were performed as previously described^9^.

### Antibodies and reagents

The following primary antibodies and reagents were used in this study: anti-ALDH1A3 (PA5-29188, 1:5,000) antibody was from Sigma-Aldrich; and anti-p65 (SC-7151, 1:200), anti-p-p65 (SC-101752, 1:200), anti-STAT3 (SC-482, 1:200) and anti-β-actin (I-19; SC-1616, 1:500) antibodies were from Santa Cruz Biotechnology. Anti-p-STAT3 (#9131, 1:1,000), anti-Histone H3 (#9715, 1:1,000), anti-β-catenin (#9562, 1:1,000), anti-β-catenin (L54E2, 1:1,000; immunofluorescence preferred, #2677, 1:200), anti-LRP6 (#2568, 1:1,000), anti-TAK1 (#5206, 1:1,000) and anti-p-TAK1 (#4531, 1:1,000) antibodies were form Cell Signaling Technology. Anti-TCF4 (#05-511, 1:500 for IB and 1:50 for IHC) and anti-ABC (#05-665, 1:1,000) antibodies were from Millipore. Anti-FZD6 (af3149, 0.2 μg ml^−1^ for IB, 10 μg ml^−1^ for IHC), ROR2 (AF2064-SP, 1 μg ml^−1^), RYK (AF4907-SP; 0.2 μg ml^−1^), Wnt4 (AF6076-SP, 1 μg ml^−1^), Wnt3a (MAB1324-SP; 2 μg ml^−1^), FZD1 (MAB11201-SP, 1 μg ml^−1^) and FZD5 (AF1617-SP; 0.5 μg ml^−1^) antibodies were from R&D Systems. Anti-APC (ab154906, 1:500), anti-FZD3 (ab56331; 1:500) and anti-NLK antibodies (ab97642, 1:1,000) were from Abcam. An anti-Ki-67 antibody (NCL-Ki67-p, 1:50) was from Leica Microsystems. An anti-Wnt5a antibody (GTX111187) was from GeneTex. The secondary antibodies (1:1,000 or 1:2,000 dilution) were from Vector Laboratories or Jackson ImmunoResearch Laboratories. Peroxidase blocking reagent was from Dako. AquaBlock was from East Coast Biologics, Inc., KN-93, 5Z-7-Oxozeaenol, Bay-117082, Cucurbitacin I (JSI-124) and an anti-CaMKII α antibody (CaMKIIα, C6974) were from Sigma-Aldrich; LGK974, ICG-001 and indomethacin were from Selleckchem; and Human Wnt3a was from R&D Systems. StemPro Accutase and Trypan blue were from Life Technologies. Cell culture media and other reagents were from Invitrogen, Sigma-Aldrich, VWR or Thermo Fisher Scientific.

### Plasmid construction and transfection

Lenti-miR-125b, miR-20b, miRZIP-125b and miRZIP-20b constructs were from System Biosciences. The pGL3-miR-125b promoter and its mutants with different deletions of TCF4-binding site and pRL-SV40 vectors were described previously^31^. A pGL3-miR-20b promoter was derived from a pGL3 plasmid, and a pGL3-miR-20b promoter mutant with a deletion of TCF4-binding site was derived from a WT pGL3-miR-20b promoter. M50 super 8 × TOPFlash (ID: 12456) and its mutation M51 super 8 × FOPFlash (ID: 12457), lentiviral-TOP-dGFP (ID: 14715), lentiviral-FOP-dGFP (ID: 14885), 7TFC (ID: 24307), TOP-GFP.mC (ID: 35491), FOP-GFP.mC (ID: 35492), Myc-tagged human full-length TCF4 pcDNA (ID: 32738), dnTCF4 (ID: 24310), pcDONR-NLK (ID: 23693) and TAK1 (ID: 23642), FZD5 (ID: 16799), puro-STAT3 shRNA (ID:26596), and shRNA for β-catenin (1248, ID:19761; 2279, ID:19762) constructs were purchased from Addgene^50^,^51^,^52^,^53^,^54^. GIPZ lentiviral shRNAs for knockdown of TCF4, FZD6, APC, FZD3, ROR2, RYK, LRP6, WNT4, WNT5A, CaMKIIα, MAP3K7, STAT3, NF-κB, and NLK were from Dharmacon. pCDH-Flag-FZD6, FZD1, FZD3, and FZD5 were generated by inserting FZD6, FZD1, FZD3, and FZD5 open reading frame (ORF) to the pCDH-CMV-MCS-EF1-Puro vector. psiCHECK2 constructs with WT FZD6 or APC were generated by inserting their 3′-untranslated region or coding-sequence region into a psiCHECK2 vector (Promega) using XhoI and NotI restriction sites. All vectors containing mutations located at the putative binding sites of miR-125b or miR-20b were generated using a QuikChange mutagenesis kit (Agilent Technologies), according to the manufacturer's instructions.

### Lentiviral production and infection

Lentiviral production and infection were carried out by co-transfecting various complementary DNA (cDNA) and packaging plasmids into 293T cells using Lipofectamine 2000 reagent according to manufacturer's instruction (#52758, Invitrogen). Forty-eight hours after transfection, the supernatants containing viruses were filtered by a 0.45-μm syringe filter (Fisher) and added into the culture media supplemented with 8 μg ml^−1^ polybrene. Forty-eight hours after the infection, transduced glioma spheres were collected and re-cultured in stem cell media. Cells with GFP expression were sorted using fluorescence-activated cell sorting (FACS). Expression of exogenous proteins or effects of shRNA knockdowns on targeting protein expression in the resultant cell populations was validated by IB and GFP expression by FACS^55^.

### Luciferase reporter assay

For the luciferase assay, 293T were seeded at 1 × 10^4^ cells per well in a 96-well plate for 24 h before transfection. Cells were co-transfected with miR-control, miR-125b or miR-20b, and psiCHECK2 constructs with WT or mutated target sequence. Twenty-four hours after transfection, the luciferase activity was measured using the Dual Luciferase Reporter Assay System (Promega). Renilla luciferase activity was normalized to corresponding firefly luciferase activity and plotted as a percentage of the controls.

### ChIP assay

ChIP assays were conducted using an EZ-ChIP kit (Millipore, Billerica, MA) according to the manufacturer's instructions. In brief, various glioma spheres were dissociated with StemPro Accutase (Thermo Fisher) into single cells. Ten million cells (1 × 10^7^) of glioma spheres were crosslinked with 1% formaldehyde for 10 min at room temperature. The crosslinking was quenched with 0.1 M glycine and washed three times with ice-cold PBS. Glioma sphere pellets were resuspended in 500 μl of lysis buffer containing protease inhibitors on ice for 10 min. Subsequently, the cell lysates were sonicated and cell debris was removed by centrifugation at 4 °C, 10,000*g* for 10 min. The samples were incubated with 60 μl Protein-A agarose for 1 h at 4 °C on a rocking platform, and then immunoprecipitated with 6 μg of an antibody against activated β-catenin (anti-ABC; MD Millipore), TCF4 (MD Millipore) or a normal IgG (control) overnight, respectively. DNA was extracted and purified for PCR using specific PCR primers (Supplementary Table), and then analysed by agarose gel electrophoresis.

### Microarray analyses

Total RNA was used for the nCounter miR platform. All sample preparation and hybridization was performed according to the manufacturer's instructions. All hybridization reactions were incubated at 65 °C for 12 h. Hybridized probes were purified and counted on the nCounter Prep Station and Digital Analyzer (NanoString) following the manufacturer's instructions. For each assay, a high-density scan was performed. For platform validation using synthetic oligonucleotides, NanoString nCounter miR raw data were normalized for lane-to-lane variation with a dilution series of six spike-in positive controls. The sum of the six positive controls for a given lane was divided by the average sum across lanes to yield a normalization factor, which was then multiplied by the raw counts in each lane to give normalized values. This set of data has been deposited into National Center for Biotechnology Information Gene Expression Omnibus website (http://www.ncbi.nlm.nih.gov/geo) with an accession code of GSE84106.

Global miRNA expression data from PN and MES glioma spheres were used for visualization in a heat map and were analysed by principal component analysis using the dChip software with statistical R package.

### Bioinformatic analyses of miRs

We first selected 20 top miRNAs that are upregulated in PN glioma spheres relative to MES glioma spheres. The GSEA was used to analyse the enrichment of those 20 miRNAs between PN and MES GBM in the TCGA data set (Supplementary Fig. 1b,c).

The StarBase v2.0 (http://starbase.sysu.edu.cn/)^27^ was used to predict potential targets and functions of miR-125b and miR-20b. ALGGEN-PROMO (http://alggen.lsi.upc.es/cgi-bin/promo_v3/promo/promoinit.cgi?dirDB=TF_8.3)^30^ was used to search for the transcription factor-binding sites in miR promoter. GSEA (http://www.broadinstitute.org/gsea/index.jsp) was used to analyse signalling pathways enrichment.

### Bioinformatic analyses of Wnt signalling enrichment

We used genes in the WNT signalling pathway obtained from the Kyoto Encyclopedia of Genes and Genomes as features to cluster TCGA PN and MES GBM. The results (Supplementary Fig. 5c) shows that tumours can be partitioned into two major subgroups, comprised of tumours from primarily the MES subtype (Grp1) or PN subtype (Grp2). Kaplan–Meier analysis shows that the survival of patients between these two subgroups are significantly different (Supplementary Fig. 5d).

### Bioinformatic analyses of TCGA GBM data set

First, we computed the mean *m* and s.d. of each miRNA expression in all TCGA GBM tumours. Then, we used these *m* and s.d. to partition tumours. A tumour was assigned into the group of high (low) expression of a miRNA or gene if its expression is larger than *m*+*r* × s.d. (less than *m*−*r* × s.d.). Finally, we used these partitions to analyse the correlation among miR-20b, miR-125b, FZD6, and TCF4, such as the low/high expression of miR-20b is associated with the high/low expression of FZD6 and low/high expression of gene TCF4. These partitions were also used to analyse cancer patients' survival.

### GBM specimens and IHC staining

All the work related to human tissues were performed in this study under the Institutional Review Board-approved protocols approved at Northwestern University in Chicago, IL, USA, and the University of Kentucky in Lexington, KY, USA, according to the National Institute of Health (NIH) guidelines. A total of 61 snap-frozen World Health Organization (WHO) grade IV GBM tissue samples were collected that informed written consent was obtained from all human participants at Northwestern University and the University of Kentucky. Histological features of all GBM specimens were confirmed by a pathologist (Dr C. Horbinski) according to WHO criteria. The small frozen fragments of tumour tissues (0.07–0.9 g) were processed for IB assays of TCF4 and FZD6 expression, or qRT–PCR analyses of miR-125b or miR-20b expression. In addition, 76 paraffin-embedded human GBM (WHO grade III and IV) specimens were collected from 2001 to 2013 at Saitama Medical University, Saitama, Japan. These clinical GBM specimens were examined and diagnosed by pathologists at Saitama Medical University.

The tissue sections of 76 clinical GBM specimens were stained with antibodies against TCF4 (1:50, 05-511, clone 6H5-3, Millipore) and FZD6 (#2963, 1:50). Nonspecific IgGs were used as negative controls. IHC was performed as previously described^55^. IHC staining was quantified as follows: 3+, positive signals in ∼50% tumour cells; 2+, positive signals in ∼25% tumour cells; 1+, positive signals in ∼5–25% tumour cells; ±, low or no positive signals in <5% tumour cells; and –, no detectable signals in all tumour cells (0%). Tumours with − or ± staining were considered as low expression and tumours with 1+ to 3+ scores were considered as high expression. Kaplan–Meier analyses for patient survival were performed using GraphPad Prism for Windows (GraphPad Software). A *χ*^2^-test was performed as previously described to examine the association between IHC staining for TCF4, FZD6 and patient survival^55^.

Mouse subcutaneous glioma sphere xenograft tumour sections were analysed by IHC (DAB or fluorescent) using anti-CD44, anti-Sox2 or anti-Ki-67 antibodies (1:200). Images were captured using an Olympus BX53 microscope equipped with an Olympus DP72 digital camera. Five random images per section of mouse brains were obtained, and percentage of positively stained cells was quantified by an Image-Pro Plus software (Version 6.2.1.491, Media Cybernetics, Inc., Bethesda, MD), and statistical analyses were performed using a GraphPad Software (GraphPad)^55^.

### Immunoblotting

Cells or tumour tissues were lysed in a RIPA buffer (50 mM Tris-HCl, pH 8.0/150 mM sodium chloride/1% NP-40/0.5% sodium deoxycholate/0.1% sodium dodecyl sulfate/2 mM EDTA) containing 1 × protease and 1 × phosphotase inhibitor cocktails (Roche). Protein samples were subjected to SDS–polyacrylamide gel electrophoresis and transferred to polyvinylidene fluoride membranes in 25 mM Tris and 192 mM glycine. Membranes were blocked with 5% nonfat dry milk in PBS, 0.05% Tween-20 and probed with indicated antibodies at desired dilutions followed by corresponding peroxidase-labelled secondary antibodies (1:200). Blots were developed with enhanced chemiluminescence (Amersham Bioscience) reaction following the manufacturer's instructions. Uncropped scans of IB presented in the main text of this study (Figs 3, 4, 5, 6) are included as the Supplementary Fig. 16 in the Supplementary Information in this paper.

### Immunofluorescent staining

Culturing glioma spheres as a monolayer was carried out using laminin as previously described^56^. In brief, stem cell medium was supplemented with laminin (10 μg ml^−1^) and used to coat tissue culture chamber slides (from Thermo Scientific) by incubating at 37 °C for 4 h before plating dissociated spheroids for monolayer growth. Cells were allowed to adhere and grow for at least 24 h before staining.

Immunofluorescent staining was performed by initially fixing the cells with a 4% paraformaldehyde solution for 20 min. Fixed cells were then washed three times with PBS-T and then blocked with AquaBlock (East Coast Bio, North Berwick, ME) for 30 min. After blocking, cells were incubated with the primary antibody (Cell Signaling, mouse anti-β-catenin antibody, catalogue# 2677, 1:200 dilution) overnight at 4 °C. Following incubation, cells were washed three times with PBS-T, stained with the 4,6-diamidino-2-phenylindole-containing mounting solution Vectashield (Vector Laboratories).

### RNA isolation and qRT–PCR

Total RNA was extracted and purified using a Qiagen RNeasy Mini kit according to the manufacturer's instructions. TaqMan miRNA assays (Applied Biosystems) were used to quantify mature miRNAs according to the supplier's instructions. The cDNA was synthesized from 500 ng of total RNA using the PrimeScript First Strand cDNA Synthesis kit (Takara). qRT–PCR was performed with the Power SYBR Green Master Mix (Life Technologies) on the Applied Biosystems StepOne Plus Real-Time Thermal Cycling Block. Results were analysed using the 2^−(ΔΔCt)^ method. Primer information used in the study can also be found in the Supplementary Table 1.

### *In vitro* cell proliferation assays

Glioma spheres were dissociated with StemPro Accutase into single cells, and cell density was quantified by counting viable, Trypan blue negative, cells using a haematocytometer. Then, cells were sorted into a 12-well plate containing 2 ml culture medium at a density of 10,000 cells per well by a BD FACSAria III flow cytometer. The cell number for living glioma spheres was counted under a Nikon inverted microscope Eclipse Ti-U at different time points using a haematocytometer.

### Analyses of sphere size and cell number

As previously described^9^, 50, 100, 200, 500 and 1,000 cells were separately sorted into each well of 96-well plates in at least eight replicates by a BD FACSAria III flow cytometer, and then cultured in the GSC medium in the presence or absence of indicated inhibitors or dimethylsulphoxide control for 6 days. Sphere size was then observed at day 6 using a Nikon inverted microscope Eclipse Ti-U equipped with a digital camera.

For glioma sphere cell counting, 1,000 cells per well were sorted into a 96-well plate in at least eight replicates by a BD FACSAria III flow cytometer and then cultured in the GSC medium in the presence or absence of indicated inhibitors or dimethylsulphoxide as control for 6 days. Single cells were dissociated from glioma spheres with StemPro Accutase. Cell number of living glioma spheres was counted under a Nikon inverted microscope Eclipse Ti-U using a haematocytometer following the addition of 50% (vol/vol) Trypan blue (Invitrogen).

### Tumorigenicity studies

All experiments using animals were performed under the Institutional Animal Care and Use Committee-approved protocol at Northwestern University in accordance with NIH and institution guidelines. Athymic (Ncr nu/nu) female mice at an age of 6–8 weeks (Taconic Biosciences) were used for all animal experiments. Patient-derived glioma spheres (5 × 10^4^ or 5 × 10^5^ cells in 2 or 5 μl PBS) were stereotactically implanted into the brains of individual mice, with five mice per group. The glioma-bearing mice were euthanized 2–6 weeks after implantation. The brains were removed, processed and analysed as we previously described^55^. For subcutaneous tumour xenograft model, 1 × 10^6^ cells of PN or MES glioma spheres in 100 or 50 μl PBS were injected into the flanks of nude mice. When palpable tumour xenografts formed, the tumour-bearing mice at every third day were treated by intraperitoneal with LGK974 (3 mg kg^−1^)^37^, BAY-117082 (10 mg kg^−1^, 5 μM)^38^ or vehicle (0.5% MC/0.5% Tween 80). Mice were euthanized when tumour sizes reached ∼1,500 mm^3^ or pathological symptoms were developed. The flank xenografted tumours were then removed, removed, embedded in OCT compound (Thermal Fisher), and stored at −80 °C. Tissues of xenografted tumours were sectioned on a cryostat in 8-μm thickness and stained with haematoxylin and eosin or IHC as described above^55^.

### Bioluminescence monitoring of subcutaneous tumour xenograft growth

Bioluminescence imaging was performed to monitor *in vivo* tumour growth using the IVIS Lumina imaging station (Caliper Life Sciences). Tumour-bearing mice were administered 300 mg kg^−1^ of D-luciferin (potassium salt, Gold Biotechnology) via intraperitoneal injection before isoflurane anaesthesia. Radiance (photons per second per square centimetre per steradian) was measured 15 min post substrate injection for each region of interest using Living Image 4.3.1 software (Caliper Life Sciences). Beginning at 1 week after subcutaneous tumour cell injection, mice were imaged weekly and at the end of the experiment.

### Statistics

Statistical analyses were performed using Microsoft Excel 2013 and GraphPad Prism version 5.00 for Windows. Analysis included one-way analysis of variance with Newman–Keuls post test and paired two-way Student's *t*-test. Survival curves were plotted by the Kaplan–Meier method and compared by log-rank and Gehan–Breslow–Wilcoxon tests as previously described^55^. *P*<0.05 was considered statistically significant.

### Data availability

DNA array data used in this study were from our published data set (GSE67089)^9^. Global miRNA expression data of PN and MES glioma spheres that were analysed by a NanoString nCounter human miRNA expression system has been deposited into National Center for Biotechnology Information Gene Expression Omnibus website (http://www.ncbi.nlm.nih.gov/geo) with an accession code of GSE84106.

All the other data supporting the findings of this study are available within the article and its supplementary information files or from the corresponding author on reasonable request.

## Additional information

**How to cite this article:** Huang, T. *et al*. A regulatory circuit of miR-125b/miR-20b and Wnt signalling controls glioblastoma phenotypes through FZD6-modulated pathways. *Nat. Commun.* **7,** 12885 doi: 10.1038/ncomms12885 (2016).

## Supplementary Material

Supplementary Information Supplementary Figures 1-16, Supplementary Table 1, Supplementary Reference
