# Supplementary material for: A regulatory circuit of miR-125b/miR-20b and Wnt signalling controls glioblastoma phenotypes through FZD6-modulated pathways
Source: Nat Commun. 2016 Oct 4;7:12885. doi: 10.1038/ncomms12885 (PMC5059456; doi:10.1038/ncomms12885)
Supplement: Supplementary Information — Supplementary Figures 1-16, Supplementary Table 1, Supplementary Reference [file ncomms12885-s1.pdf]

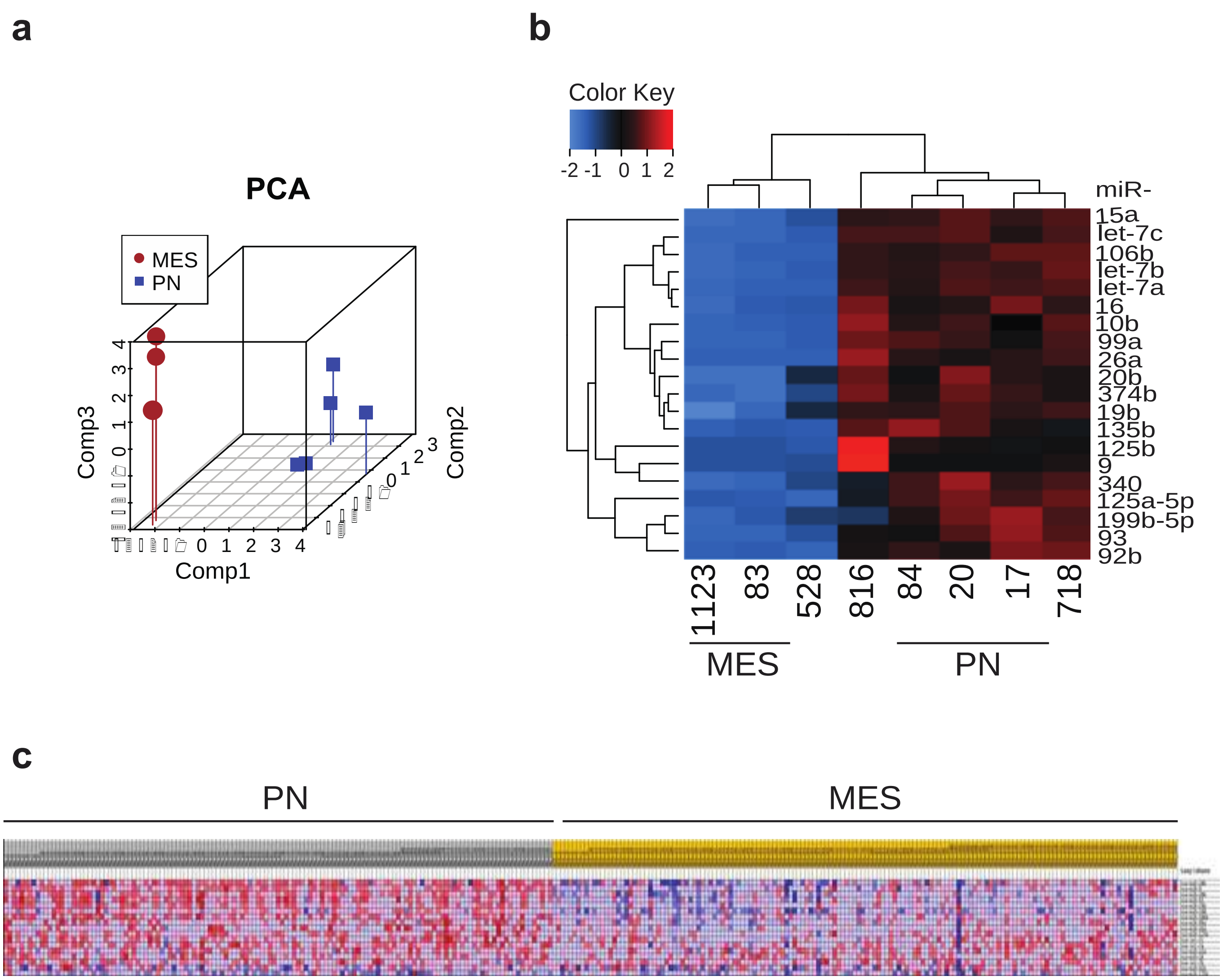

Supplementary Figure 1 (Related to Figure 1) (**a**) Principal component analysis (PCA) using Cluster 3.0 software shows PN miRNA expression profile clusters glioma PN spheres 84, 17, 20, and MES clusters 83, 1123. (**b**) The top 20 miRNAs that are enriched in PN glioma spheres compared to MES glioma spheres. Heat maps generated using Java Tree view 1.1.6 using Cluster 3.0 software as shown in Figure 1A. (**c**) Analysis of miRNA expression in GBM of TCGA dataset. 20 miRNAs with top-ratios between expression averages of PN and MES glioma spheres also cluster in PN and MES clinical GBM.

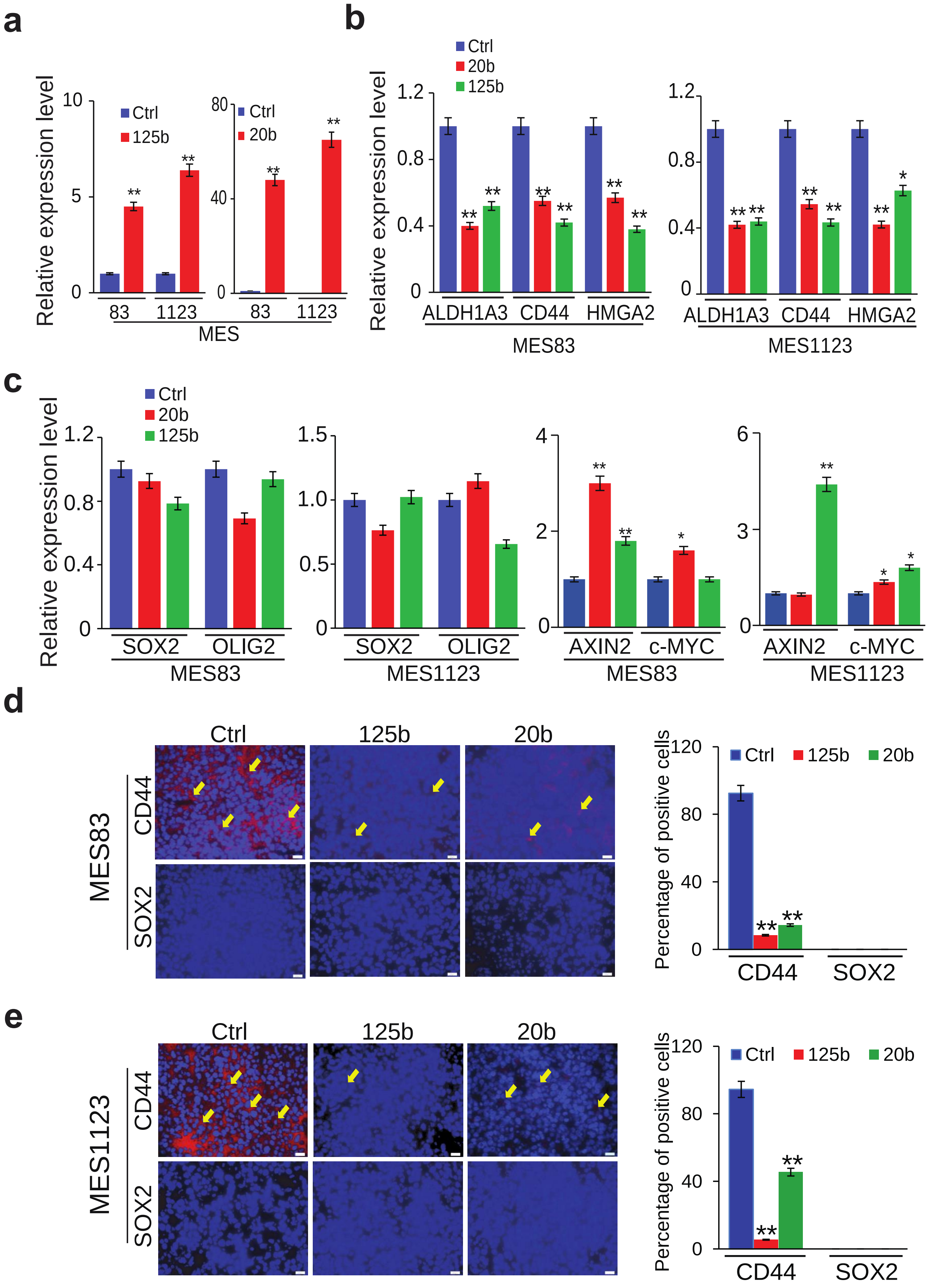
a
Relative expression level
Ctrl
125b
20b
83
1123
MES
b
ALDH1A3
CD44
HMGA2
MES83
MES1123
c
SOX2
OLIG2
AXIN2
c-MYC
d
MES83
125b
Percentage of positive cells
e
MES1123

Supplementary Figure 2 (Related to Figure 2). Overexpression of miR-125b and miR-20b suppressed MES-associated genes expression and increased Wnt-related genes in glioma spheres. qRT-PCR analyses were performed to determine the expression levels of miR-125b and miR-20b (**a**), MES-associated genes *ALDH1A3*, *CD44*, and *HMGA2* (**b**), and PN-associate genes *SOX2* and *OLIG2*, as well as Wnt-related genes Axin2 and c-Myc **(c**), in MES 83 and 1123 glioma spheres that separately overexpress miR-125b, miR-20b, or a control miRNA. (**d**) and (**e**), immunofluorescent IHC analyses of protein expression of CD44 and SOX2 in brain tumor xenografts established at 16 days post-implantation by MES 83 (**d**) and 1123 (**e**) spheres that separately overexpress miR-125b, miR-20b, or a control miRNA. Scale bar: 20 µm. Yellow arrows indicate positively stained cells. Right bar graphs, quantitation of positively stained cells. Data were collected from 3 separate tumors per group and 5 images per tumor. Error bars (s.d.) represent data of triplicate samples for each set of experiments. *p <0.05, **p <0.01, paired two-way Student t-test. Data are representative from three independent experiments with similar results.

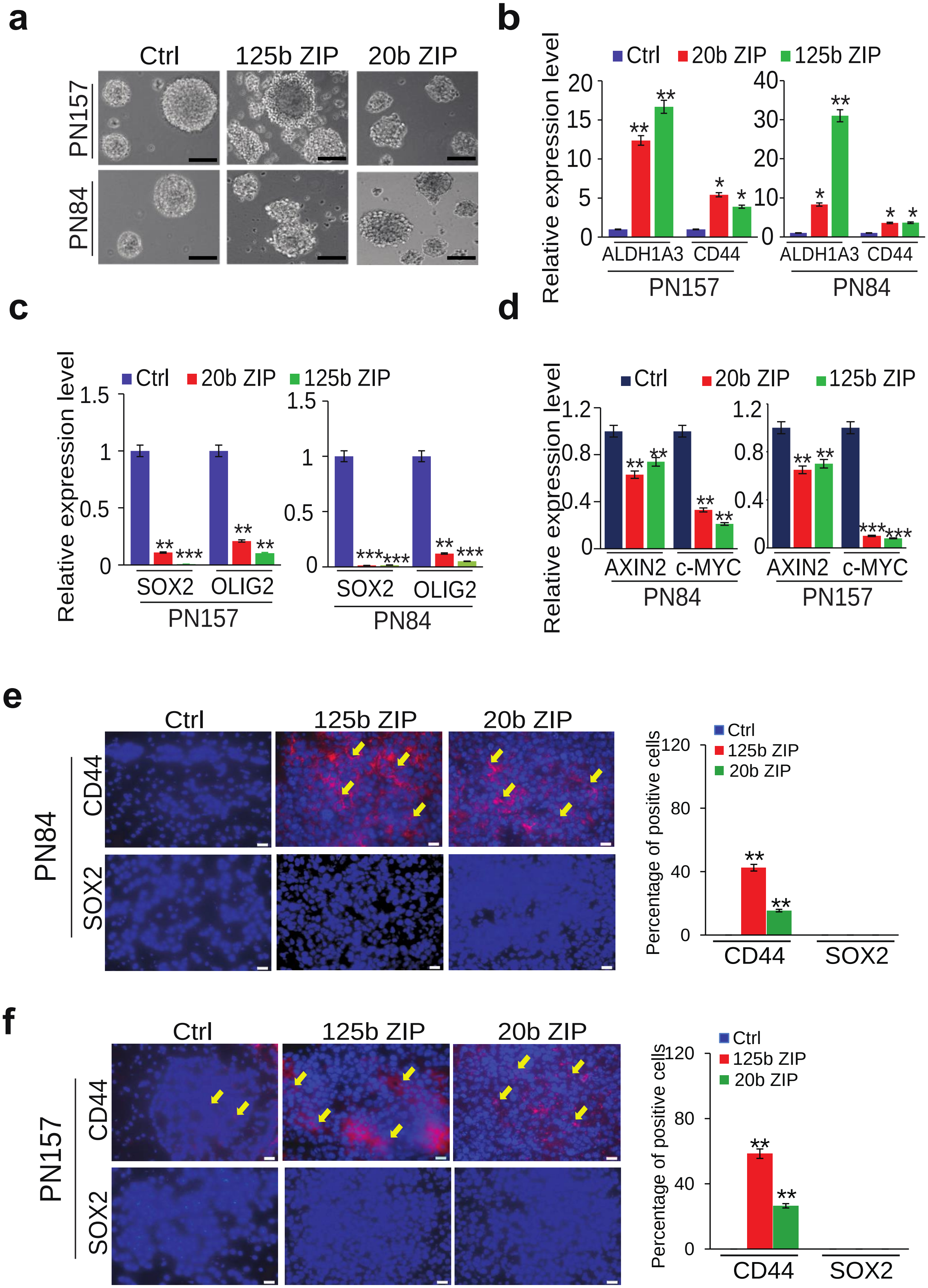
a
Ctrl
125b ZIP
20b ZIP
PN157
PN84
b
Ctrl
20b ZIP
125b ZIP
Relative expression level
ALDH1A3
CD44
PN157
PN84
c
Ctrl
20b ZIP
125b ZIP
Relative expression level
SOX2
OLIG2
PN157
PN84
d
Ctrl
20b ZIP
125b ZIP
Relative expression level
AXIN2
c-MYC
PN84
PN157
e
Ctrl
125b ZIP
20b ZIP
PN84
CD44
SOX2
Percentage of positive cells
f
Ctrl
125b ZIP
20b ZIP
PN157
CD44
SOX2
Percentage of positive cells

Supplementary Figure 3 (Related to Figure 2) Inhibition of miR-125b and miR-20b induced MES-associated gene expression, but suppressed Wnt-related genes in PN glioma spheres. (**a**) Representative images of neurospheres of PN 157 and PN 84 that separately express miR-125 ZIP, miR-20b ZIP, or a control miRZIP. Scale bar: 50 µm. (**b**) MES-associated genes *ALDH1A3* and *CD44*, (**c**) PN-associated genes *SOX2* and *OLIG2,* and (**d**) Wnt-related genes *AXIN2* and *c-MYC* were analyzed using qRT-PCR analyses in PN 157 and 84 that express miR-20b ZIP, miR-125 ZIP or a control miRNA ZIP. β-actin was used as an internal control. (**e**), (**f**), immunofluorescent IHC analyses of protein expression of CD44 and SOX2 in brain tumor xenografts established by PN 84 (**e**) and 157 (**f**) spheres that separately overexpress miR-125 ZIP, miR-20b ZIP, or a control miRZIP. Both control PN 84 and 157 glioma spheres failed to form sizable brain tumor xenografs on 12 (PN 84) or 15 (PN 157) days post-implantation. Thus it was difficult to detect PN-associated Sox2 protein expression in these brain sections (**e** and **f**, lower panels and bar graphs). Scale bar: 20 µm. Yellow arrows indicate positively stained cells. Right bar graphs, quantitation of positively stained cells. Data were collected from 3 separate tumors per group and 5 images per tumor. Error bars (s.d.) represent data of triplicate samples for each set of experiments. *p <0.05, **p <0.01, ***p <0.001, paired two-way Student t-test. Data are representative from three independent experiments with similar results.

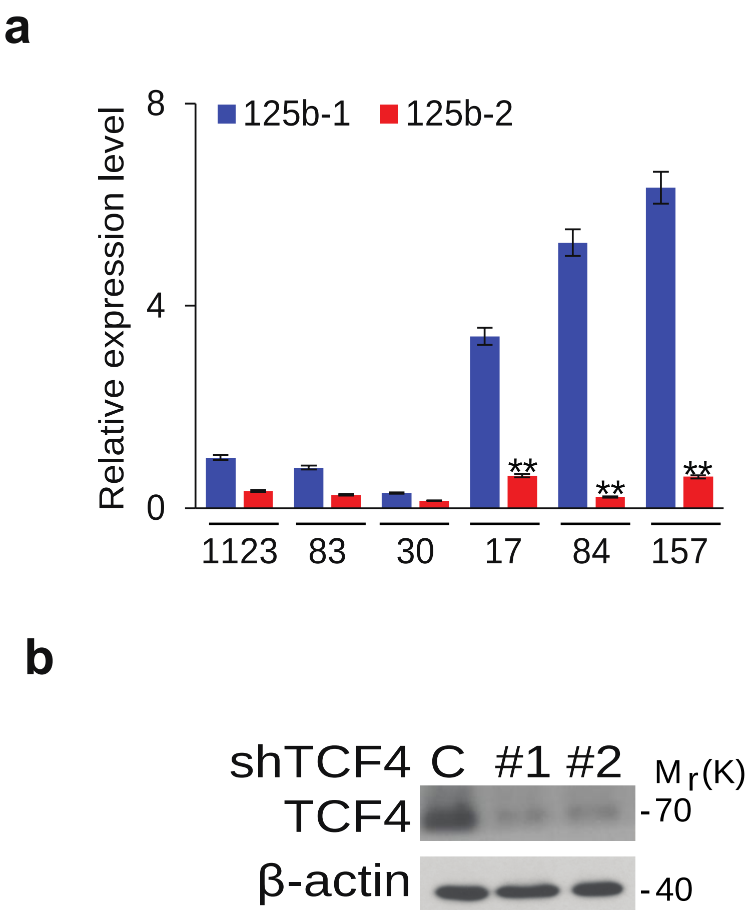

Supplementary Figure 4 (Related to Figure 3) (**a**) The pre-miR-125b-1 on chromosome hsa11 is a major pre-miR that contributes to mature miR-125b expression in glioma spheres. qRT-PCR analyses were performed to determine the expression levels of pre-miR-125b-1 and pre-miR-125b-2 in indicated glioma spheres. (**b**) IB analysis of TCF4 protein on PN expressing a control shRNA and two TCF4 specific shRNAs. Error bars (s.d.) represent data of triplicate samples for each set of experiments. **p <0.01, paired two-way Student t-test. Data are representative from two independent experiments with similar results.

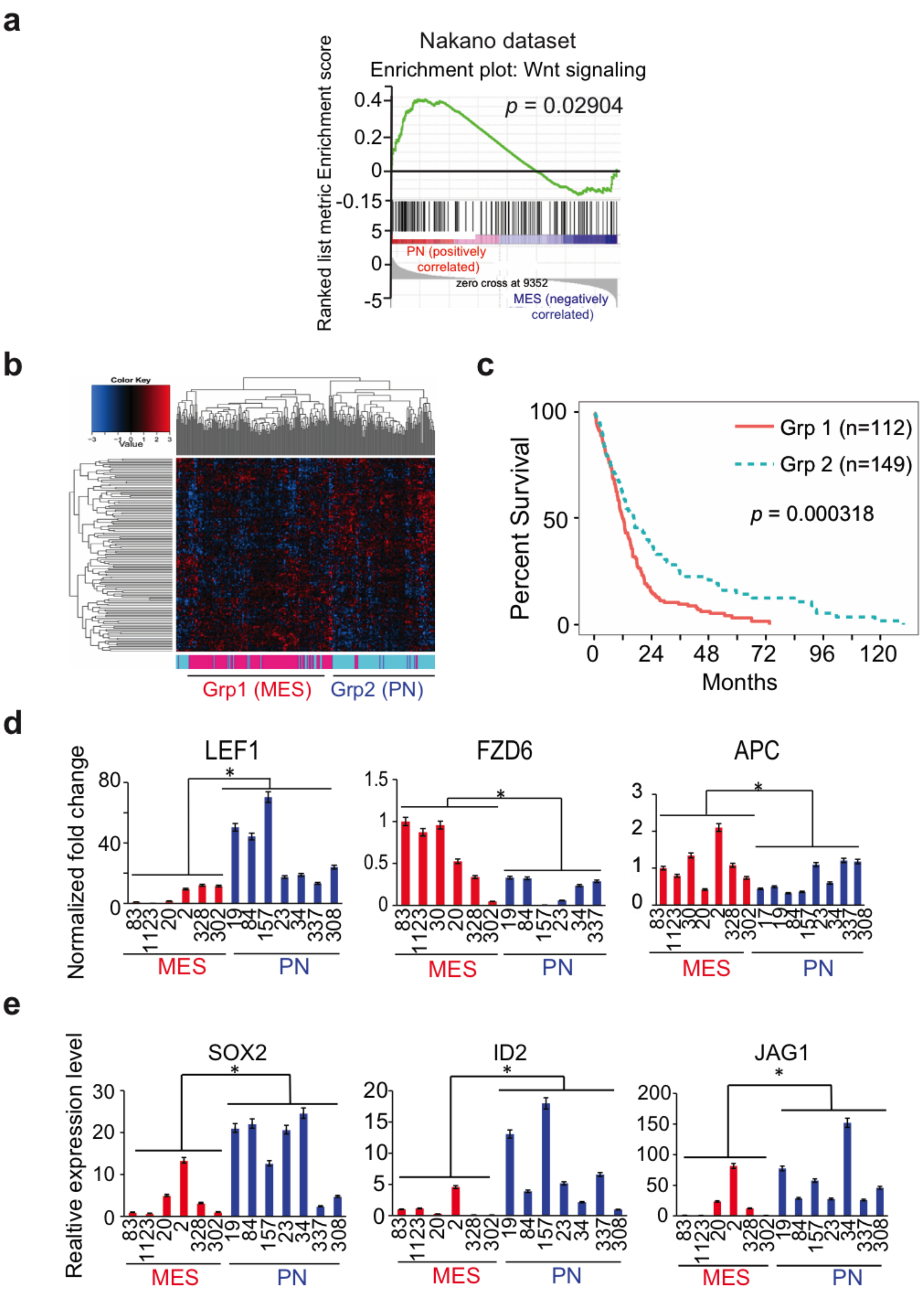
a
Nakano dataset
Enrichment plot: Wnt signaling
p = 0.02904
Ranked list metric Enrichment score
0.4
0.2
0
-0.15
5
0
-5
PN (positively correlated)
zero cross at 9352
MES (negatively correlated)
b
Color Key
Value
Grp1 (MES)
Grp2 (PN)
c
Grp 1 (n=112)
Grp 2 (n=149)
p = 0.000318
Percent Survival
Months
d
LEF1
FZD6
APC
Normalized fold change
MES
PN
e
SOX2
ID2
JAG1
Realtive expression level
MES
PN

f

	PN			MES	
	84	157	17	83	1123
WNT1	11.9	11.6	11.9	11.7	12.7
WNT2	11.7	12.0	11.5	11.7	12.9
WNT2B	16.3	16.8	16.0	19.8	21.9
**WNT3**	89.5	94.5	152.5	183.7	186.4
WNT3A	15.3	15.5	14.7	16.5	17.5
WNT4	22.2	20.5	20.2	22.4	24.6
WNT5A	21.6	24.3	14.6	18.3	18.5
WNT5B	23.9	22.9	26.5	47.5	67.2
WNT6	19.3	20.3	19.6	21.0	25.0
WNT7A	25.6	25.5	25.7	22.5	27.3
WNT7B	53.8	32.7	44.8	18.0	20.9
WNT8A	12.7	13.2	12.6	12.0	13.5
WNT8B	9.5	11.0	9.6	9.8	10.1
WNT9A	14.7	14.1	14.7	14.9	16.0
WNT9B	27.6	26.0	26.6	24.9	27.2
WNT10A	13.7	14.1	14.3	13.5	15.8
WNT10B	59.1	59.7	58.0	71.1	72.0
WNT11	12.5	11.7	12.1	14.1	14.0
WNT16	9.2	9.6	8.9	9.8	10.0

	PN			MES	
	84	157	17	83	1123
**FZD1**	72.1	82.0	320.9	53.2	40.3
**FZD2**	145.8	138.2	155.1	146.5	530.6
**FZD3**	306.4	348.7	201.3	24.4	18.9
FZD4	23.5	23.2	17.3	19.6	20.0
**FZD5**	244.9	149.3	70.5	36.7	34.1
**FZD6**	13.3	13.1	62.7	271.2	410.1
FZD7	68.8	56.4	55.3	154.8	144.1
**FZD8**	179.6	132.5	131.9	456.6	66.3
FZD9	17.0	16.2	15.3	16.2	17.3
FZD10	15.3	15.4	14.5	14.8	16.1
**LRP6**	311.6	343.8	294.7	82.6	87.9
ROR2	15.3	16.2	15.4	35.3	19.0
**RYK**	594.6	563.8	581.4	709.4	606.4

g

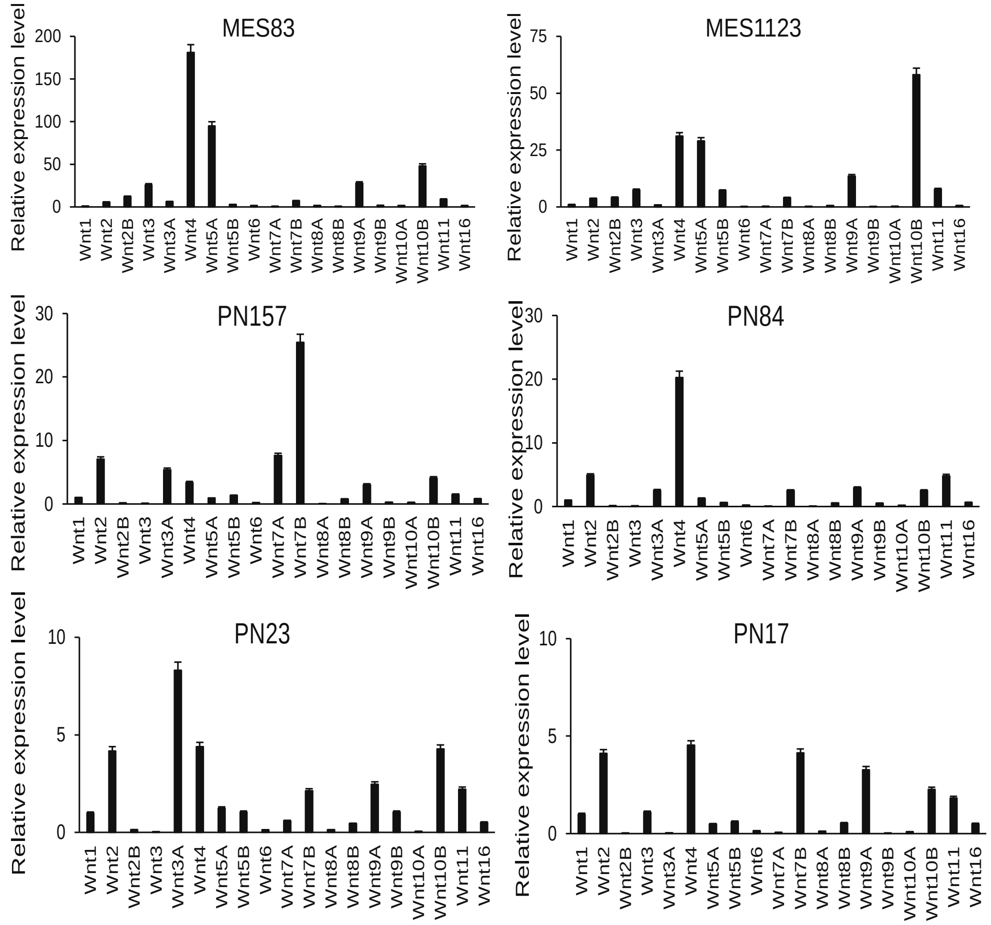

h

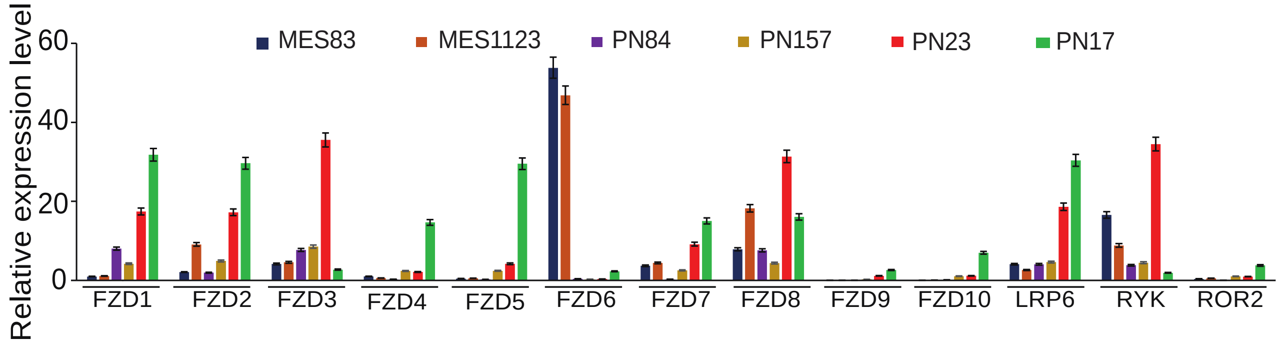

i

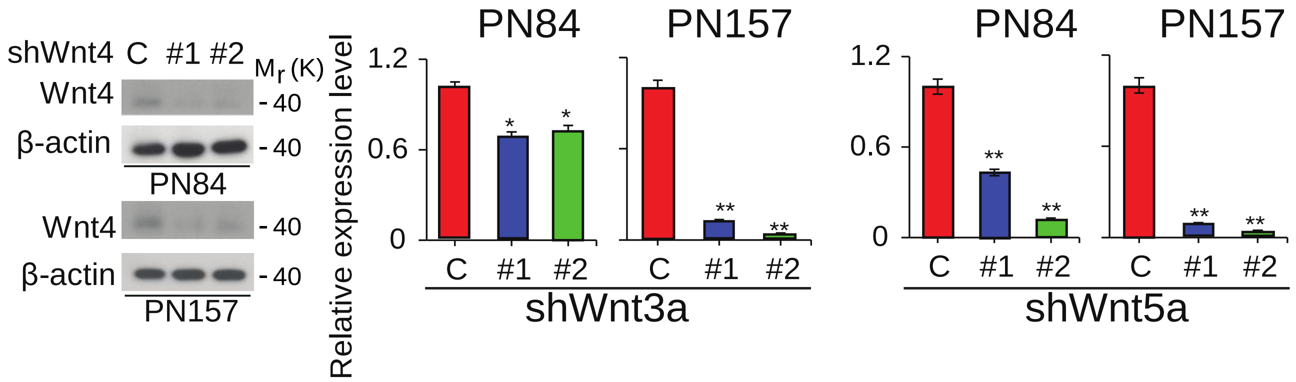

j

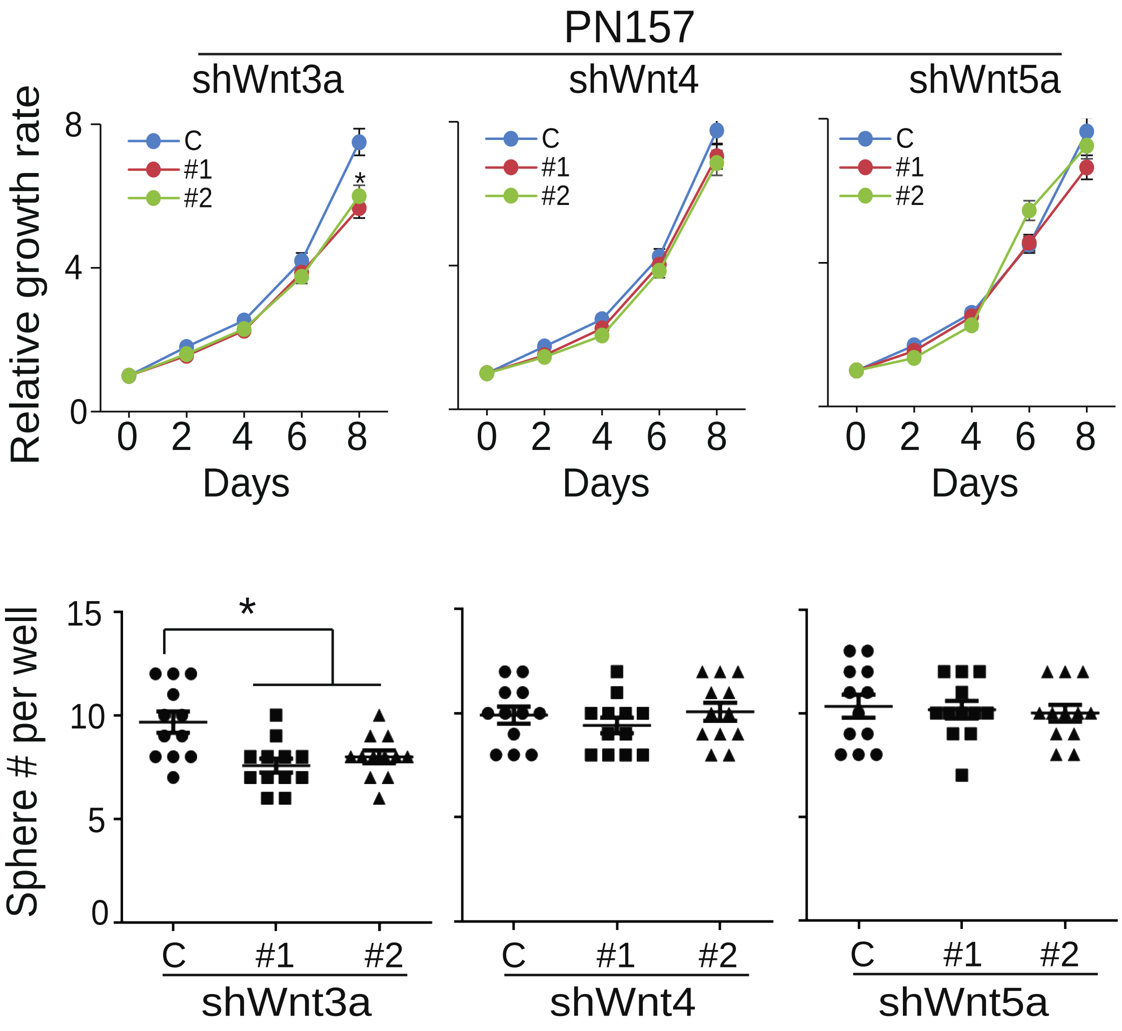

Supplementary Figure 5 (Related to Figure 4) Wnt signaling activity is active in PN but not MES glioma spheres. (**a**) Gene Set Enrichment Analysis (GSEA) of data sets of Nakano (GEO# GSE67089)[1] for enrichment of the canonical Wnt pathway between PN and MES GBM. (**b**) Analysis of TCGA dataset for enrichment of Wnt signaling pathway (KEGG) clustered group 1 (Grp1, most are MES) and group 2 (Grp2, most are PN) GBM tumors. (**c**) Kaplan-Meier survival

analysis of TCGA data (Wnt signaling pathway enrichment) of two patient subgroups (Grp1-MES vs Grp2-PN) obtained from (**b**). (**d,e**) qRT-PCR analyses were performed to validate expression of LEF1, FZD6 and APC, and canonical Wnt signaling target genes *SOX2*, *ID2* and *JAG1* in indicated PN and MES glioma spheres. (**f**) cDNA array data of Wnt ligands, FZDs, LRP6, ROR2, and RYK (GEO# GSE67089)[1]. (**g**,**h**) qRT-PCR analyses were performed to validate the expression of 19 WNT ligands, 10 FZDs, LRP6, ROR2, and RYK in PN and MES spheres. (**i**) IB analysis of WNT4 protein on PN 84 and 157 expressing a control shRNA and *WNT4* specific shRNAs. Right panels, due to incapability of detecting WNT3a and WNT5a proteins using IB with commercial available antibodies, qRT-PCR analyses were used to validate mRNA expression of *WNT3a* and *WNT5a* in PN 84 and 157 expressing a control shRNA vector and specific shRNAs targeting WNT3a or WNT5a, respectively. (**j**) Cell proliferation (upper panels) and neural sphere formation assays (lower panels) for PN157 expressing a control shRNA or specific shRNAs targeting *WNT3a*, *WNT4* or *WNT5a*, respectively. Error bars (s.d.) represent data of triplicate samples for each set of experiments. *p <0.05, **p <0.01, ***p <0.001, paired two-way Student t-test. Data are representative from three independent experiments with similar results.

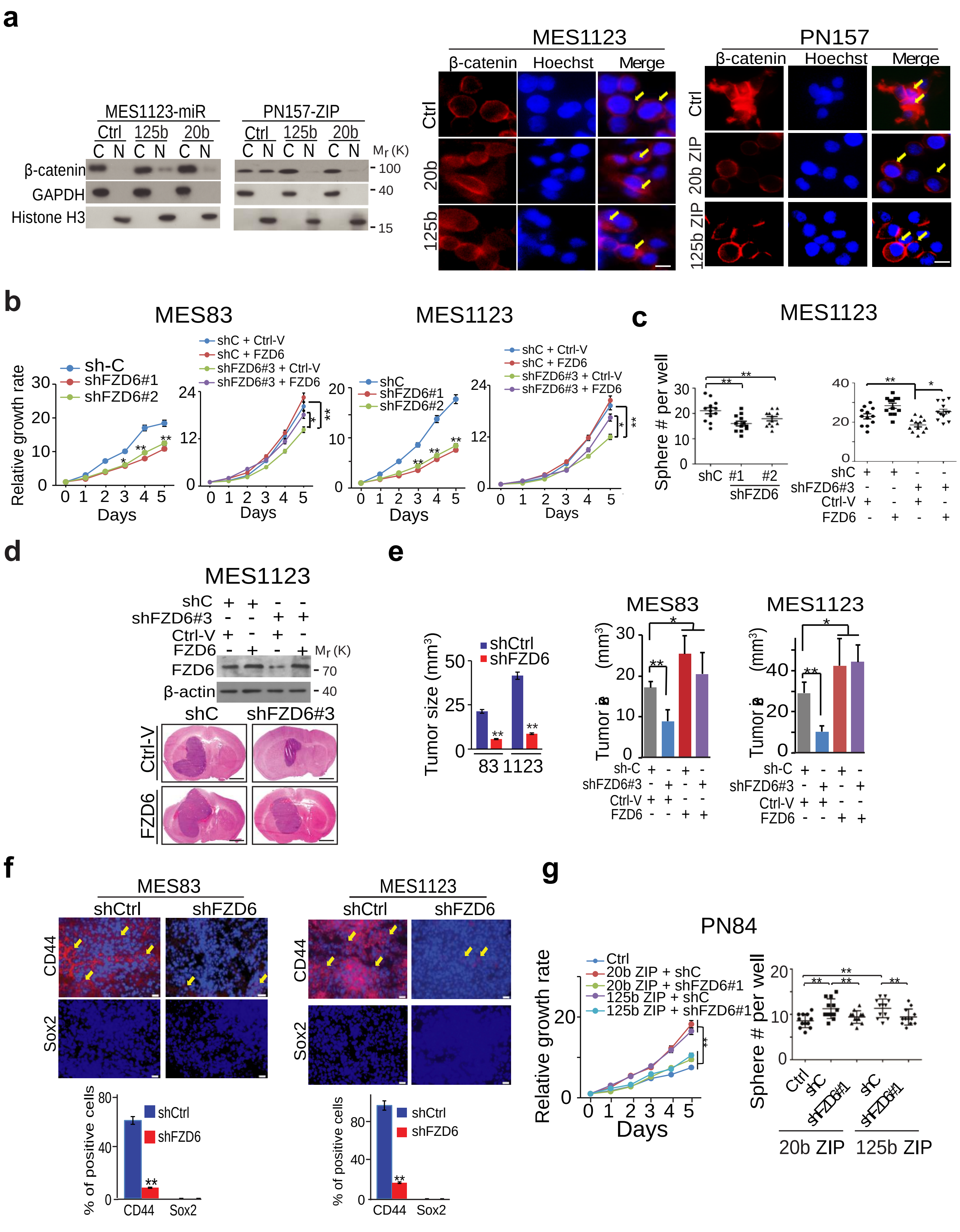
a
MES1123-miR
Ctrl 125b 20b
C N C N C N
PN157-ZIP
Ctrl 125b 20b
C N C N C N
β-catenin
GAPDH
Histone H3
Mr (K)
100
40
15
MES1123
β-catenin Hoechst Merge
Ctrl
20b
125b
PN157
β-catenin Hoechst Merge
Ctrl
20b ZIP
125b ZIP
b
MES83
sh-C
shFZD6#1
shFZD6#2
Relative growth rate
Days
shC + Ctrl-V
shC + FZD6
shFZD6#3 + Ctrl-V
shFZD6#3 + FZD6
MES1123
shC
shFZD6#1
shFZD6#2
c
MES1123
Sphere # per well
shC #1 #2
shFZD6
shC
shFZD6#3
Ctrl-V
FZD6
d
MES1123
shC
shFZD6#3
Ctrl-V
FZD6
Mr (K)
FZD6
β-actin
70
40
shC shFZD6#3
Ctrl-V
FZD6
e
shCtrl
shFZD6
Tumor size (mm³)
83 1123
MES83
MES1123
sh-C
shFZD6#3
Ctrl-V
FZD6
f
MES83
shCtrl shFZD6
CD44
Sox2
MES1123
shCtrl shFZD6
% of positive cells
CD44 Sox2
g
PN84
Ctrl
20b ZIP + shC
20b ZIP + shFZD6#1
125b ZIP + shC
125b ZIP + shFZD6#1
Relative growth rate
Days
Sphere # per well
Ctrl
shC
shFZD6#1
20b ZIP 125b ZIP

Supplementary Figure 6 (Related to Figure 5) miR-20b/miR-125b affected the cellular localization of β-catenin and inhibition of FZD6-impeded growth, self-renewal, and tumor growth of MES glioma spheres, (**a**) Left panels, IB analyses. Protein levels of β-catenin in cytoplasmic (C) and nucleus (N) were examined by IB using the indicated antibodies in miR-transduced MES 1123, or miRZIP-transduced PN 157. GAPDH and histone H3 were used as cytoplasmic and nuclear protein markers, respectively. Right panels, immunofluorescence staining of subcellular β-catenin localization in the indicated cells using indicated antibodies. Scale bar, 20 µm. (**b**) Cell proliferation of MES83 and 1123 expressing shFZD6s or shC, with or without FZD6 rescued expression (#1/#2 targeting FZD6 ORF, while #3 targeting FZD6 3'UTR). (**c**) Neurosphere formation of MES 1123 expressing shFZD6s or shC, with or without FZD6 rescued expression. (**d**) Knockdown of FZD6 #3 inhibited tumor growth of MES 1123 GBM xenografts in the brain, whereas re-expression of FZD6, but not a vector control, rescues shFZD6 #3-inhibited tumor growth ($n = 5$). (**e**) Tumor areas of indicated group in (**d**) and in Fig. 5g were quantified. Scale bar: 1.0 mm. (**f**) Immunofluorescent IHC analyses of protein expression of CD44 and Sox2 in brain tumor xenografts established by MES 83 and 1123 glioma spheres that separately express shRNA for FZD6 (shFZD6#1), or a control shRNA (shCtrl). Scale bar: 20 µm. Yellow arrows indicate positively stained cells. Bar graphs below show quantitation of positively stained cells. Data were collected from three separate tumors per group and five images per tumor. (**g**) Cell proliferation (left) and neurosphere formation (right) of PN 84 expressing miR-20b ZIP, miR-125b ZIP, or a control miRZIP with or without FZD6 knockdown. Error bars (s.d.) represent data of triplicate samples for each set of experiments. *$p < 0.01$, **$p < 0.01$, paired two-way Student t-test. Data are representative from three independent experiments with similar results.

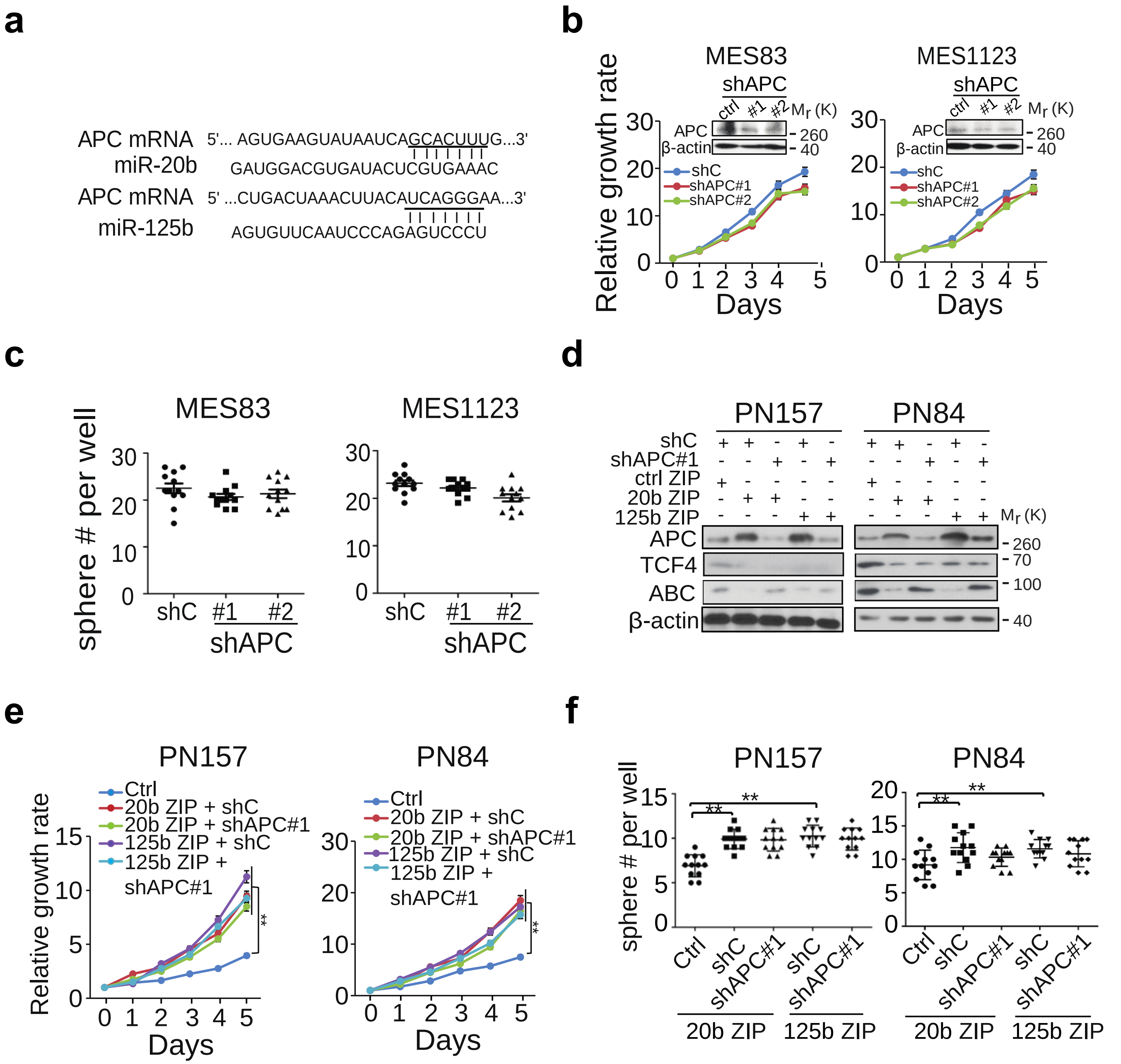

Supplementary Figure 7 (Related to Figure 5) (**a**) Targeting sites of miR-20b and miR-125b in 3'-UTR downstream of the coding sequences of *APC* gene. Nucleotides complementary to the seed sequence of miR-20b and miR-125b are underlined. (**b,c**) Inhibition of APC exhibited negligible effects on cell growth and self-renewal of glioma spheres. Cell proliferation (**b**) and neurosphere formation assay (**c**) were performed in MES 83 and 1123 expressing a control shRNA or APC specific shRNAs. Inserts: IB of shRNA knockdown of APC in glioma spheres using the indicated antibodies. (**d**) IB analysis. Effects of shRNA knockdown of APC with or without expression of miR-20b ZIP, miR-125b-ZIP, or a control Zip on expression of TCF4 and active β-catenin (ABC) in PN 157 or 84. (**e**,**f**), Effects of inhibition of APC (shRNA knockdown) or miRs (miRZIP inhibition) on cell proliferation (**e**) and neurosphere formation (**f**) of PN 157 and 84. Error bars (s.d.) represent data of triplicate samples for each set of experiments. **p <0.01, paired two-way Student t-test. Data are representative from three independent experiments with similar results.

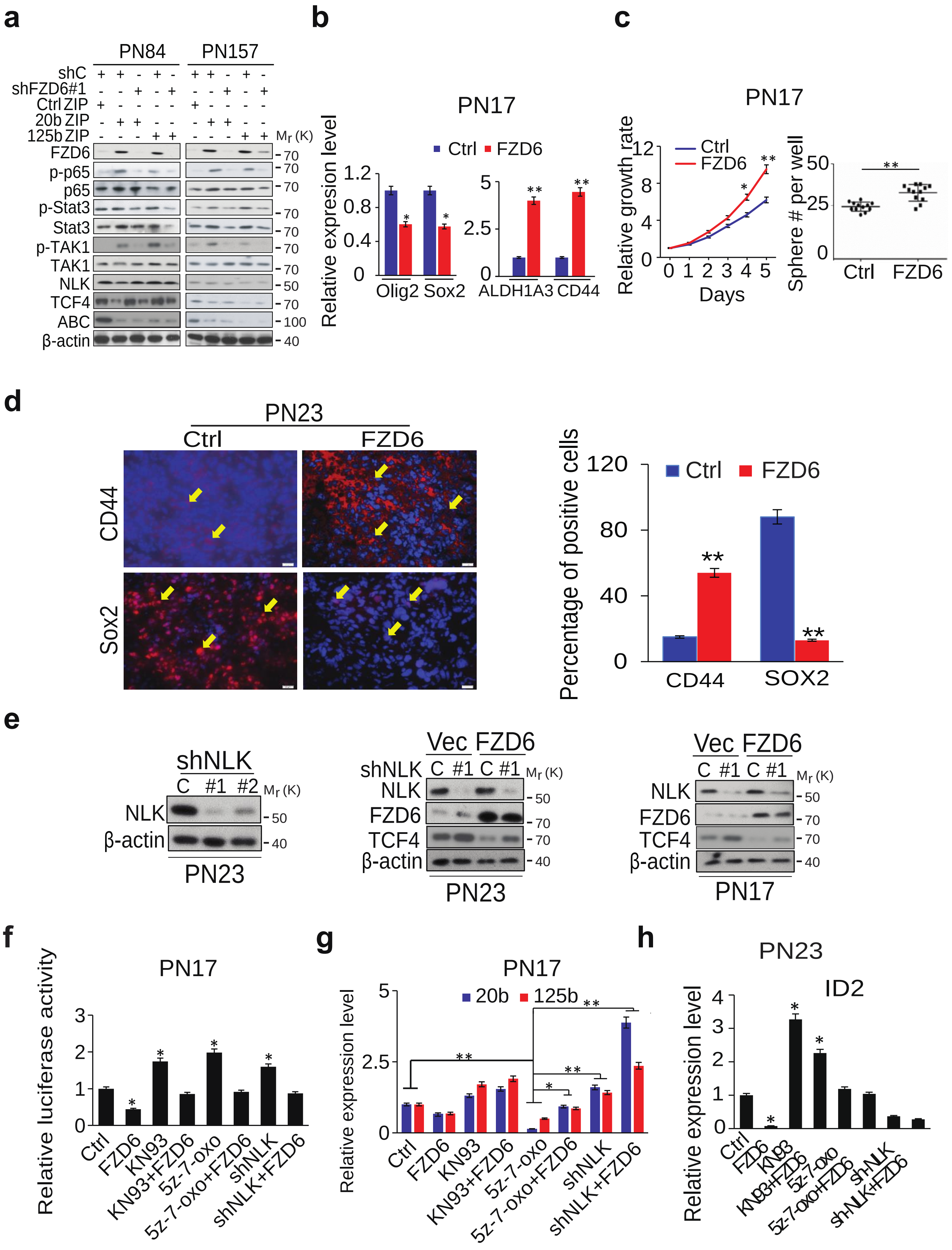
a
PN84
PN157
shC
shFZD6#1
Ctrl ZIP
20b ZIP
125b ZIP
Mr (K)
FZD6
p-p65
p65
p-Stat3
Stat3
p-TAK1
TAK1
NLK
TCF4
ABC
β-actin
70
70
70
70
70
70
70
50
70
100
40
b
PN17
Ctrl
FZD6
Relative expresion level
1.2
0.8
0.4
0
Olig2
Sox2
5
2.5
0
ALDH1A3
CD44
c
PN17
Relative growth rate
12
8
4
0
Ctrl
FZD6
0 1 2 3 4 5
Days
Sphere # per well
50
25
0
Ctrl
FZD6
d
PN23
Ctrl
FZD6
CD44
Sox2
Percentage of positive cells
120
80
40
0
Ctrl
FZD6
CD44
SOX2
e
shNLK
C #1 #2
Mr (K)
NLK
β-actin
50
40
PN23
Vec FZD6
shNLK C #1 C #1
Mr (K)
NLK
FZD6
TCF4
β-actin
50
70
70
40
PN23
Vec FZD6
C #1 C #1
Mr (K)
NLK
FZD6
TCF4
β-actin
50
70
70
40
PN17
f
PN17
Relative luciferase activity
3
2
1
0
Ctrl
FZD6
KN93
KN93+FZD6
5z-7-oxo
5z-7-oxo+FZD6
shNLK
shNLK+FZD6
g
PN17
20b
125b
Relative expression level
5
2.5
0
Ctrl
FZD6
KN93
KN93+FZD6
5z-7-oxo
5z-7-oxo+FZD6
shNLK
shNLK+FZD6
h
PN23
ID2
Relative expression level
4
3
2
1
0
Ctrl
FZD6
KN93
KN93+FZD6
5z-7-oxo
5z-7-oxo+FZD6
shNLK
shNLK+FZD6

Supplementary Figure 8, (Related to Figure 6) FZD6 regulates properties of glioma spheres and miR-125b and miR-20b expression through the CaMKII-TAK1-NLK signaling. (**a**) IB analysis using indicated antibodies on PN 84 and 157 that express miR-20b ZIP or miR-125b ZIP with or without knockdown of FZD6. shC, a control scrambled shRNA. Ctrl: control. (**b**) qRT-PCR analysis of PN-associated markers (*Olig2* and *Sox2*) and MES-associated markers (*ALDH1A3* and *CD44*). (**c**) Cell proliferation (left) and sphere formation assays (right) of PN 17 spheres with FZD6 overexpression. (**d**) Immunofluorescent IHC analyses of protein expression of CD44 and Sox2 in brain tumor xenografts established by PN 23 spheres that express FZD6, or a control vector (Ctrl). Scale bar: 20 µm. Yellow arrows indicate positively stained cells. Right bar graph is the quantitation of positively stained cells. Data were collected from 3 separate tumors per group and 5 images per tumor. (**e**) IB analysis using indicated antibodies on PN 23 and 17 spheres with or without NLK knockdown and FZD6 overexpression. (**f**) Luciferase reporter assay using a lentivirus TOP Flash/FOP Flash to quantify relative Wnt signaling activity on PN 17 spheres that overexpress FZD6 or a control vector with or without treatments of CaMKII Inhibitor KN93 (5 µM), TAK1 inhibitor 5Z-7-oxo (5 µM), or shNLK lentivirus. (**g,h**) qRT-PCR analysis of the relative expression of miR-20b, miR-125b and a Wnt target gene ID2 in PN 17 (**g**) or 23 (**h**) spheres that overexpress FZD6 or a control vector, with or without various treatments as indicated. Error bars (s.d.) represent data of triplicate samples for each set of experiments. . *p <0.05, **p <0.01, paired two-way Student t-test. Data are representative from three independent experiments with similar results.

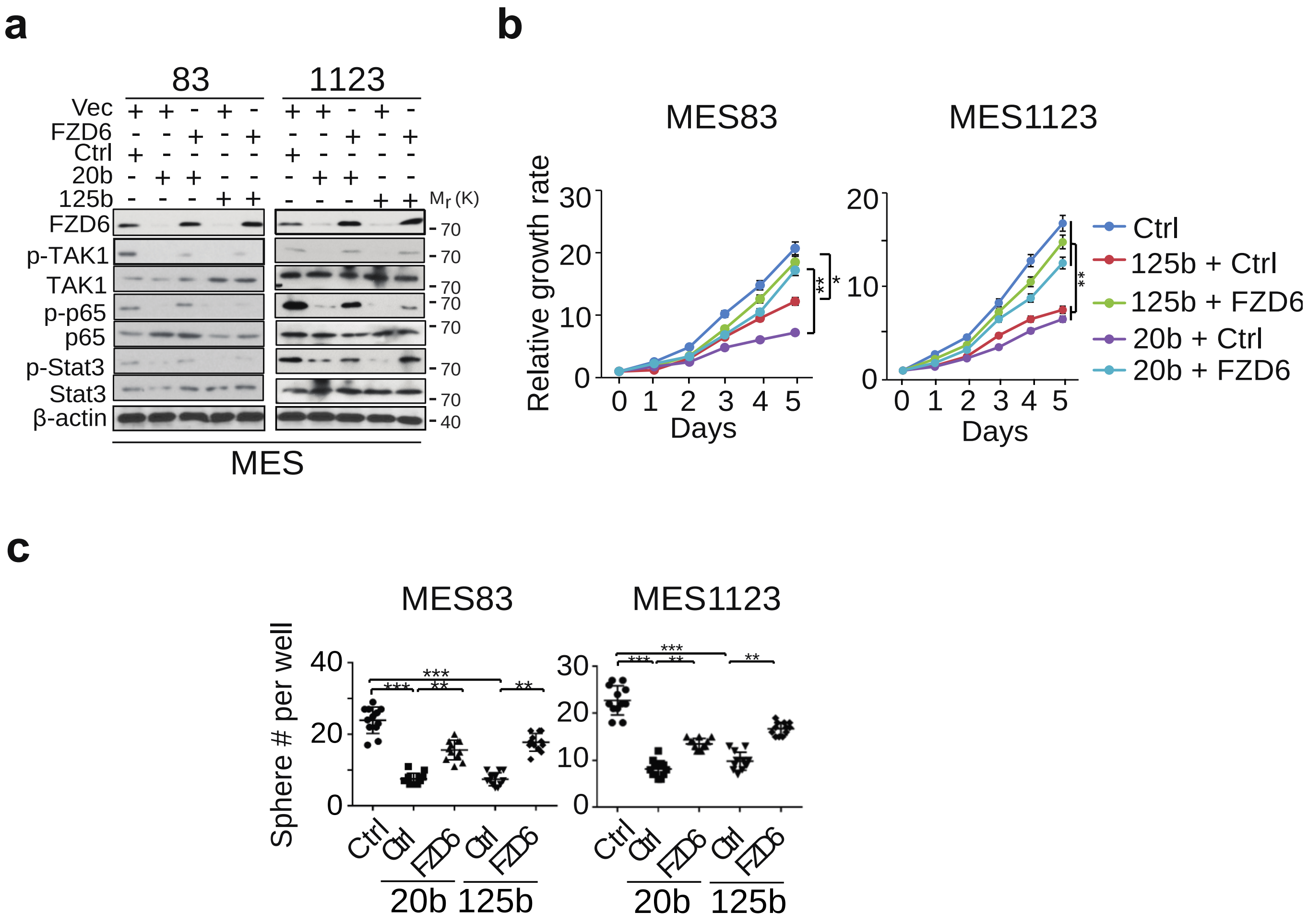

Supplementary Figure 9 (Related to Figure 6) miR-20b, miR-120b and FZD6 regulate downstream effectors of FDZ6, TAK1, NF-κB and STAT3 and self-renewal, and cell growth of MES glioma spheres. (**a**) IB analysis using indicated antibodies. (**b**) Cell proliferation and (**c**) neurosphere formation assay of MES 83 and 1123 that expressed miR-20b, miR-125b or a control miRNA with or without FZD6 overexpression. Error bars (s.d.) represent data of triplicate samples for each set of experiments. . *p <0.05, **p <0.01, ***p <0.001, paired two-way Student t-test. Data are representative from three independent experiments with similar results.

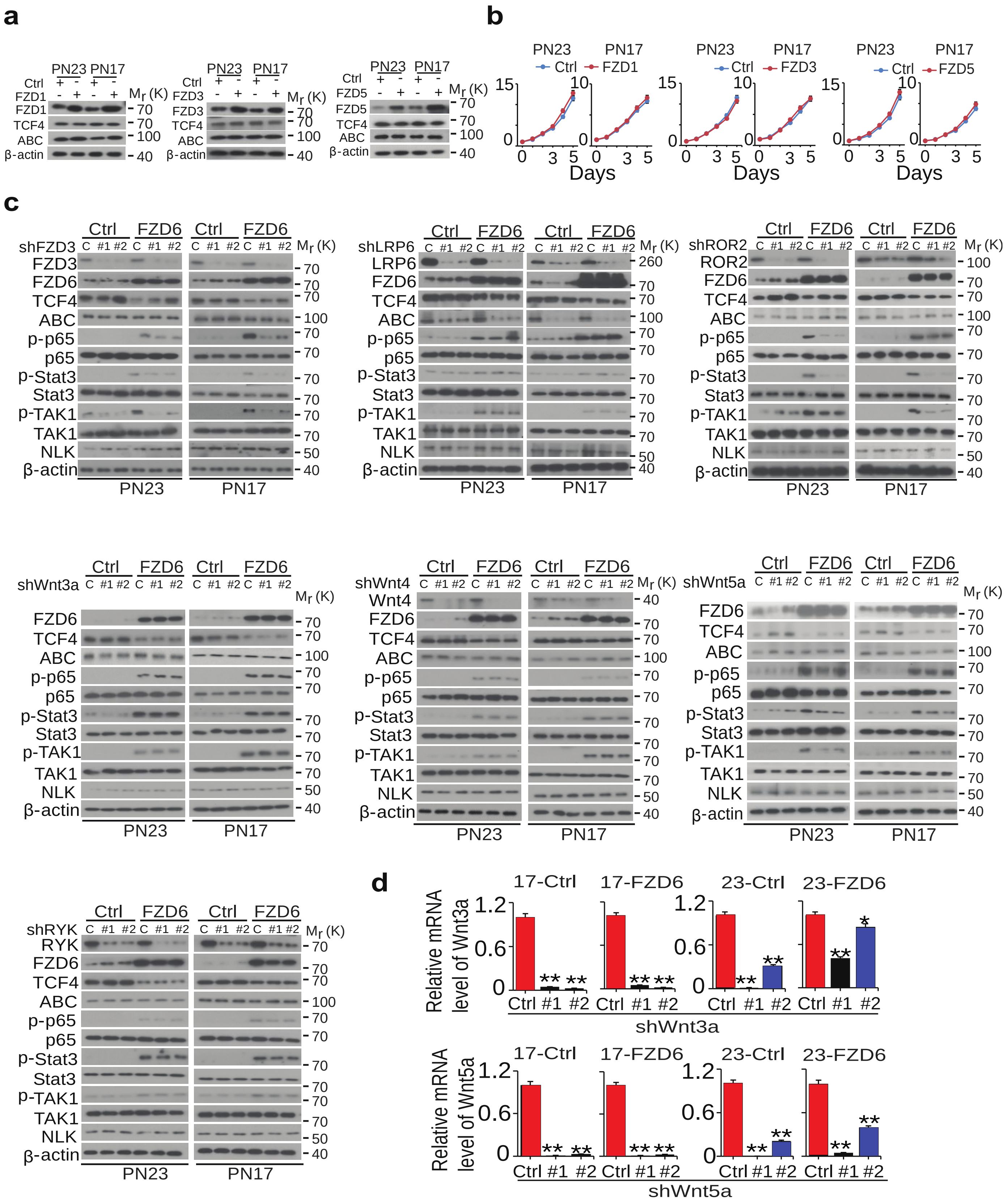
a
PN23 PN17
Ctrl
FZD1
FZD3
FZD5
TCF4
ABC
β-actin
Mr (K)
70
100
40
b
PN23
PN17
Ctrl
FZD1
FZD3
FZD5
15
10
0
3
5
Days
c
Ctrl
FZD6
shFZD3
shLRP6
shROR2
shWnt3a
shWnt4
shWnt5a
shRYK
C #1 #2
FZD3
LRP6
ROR2
Wnt4
RYK
FZD6
TCF4
ABC
p-p65
p65
p-Stat3
Stat3
p-TAK1
TAK1
NLK
β-actin
Mr (K)
260
100
70
50
40
PN23
PN17
d
17-Ctrl
17-FZD6
23-Ctrl
23-FZD6
Relative mRNA level of Wnt3a
Relative mRNA level of Wnt5a
1.2
0.6
0
Ctrl #1 #2
shWnt3a
shWnt5a
**
*

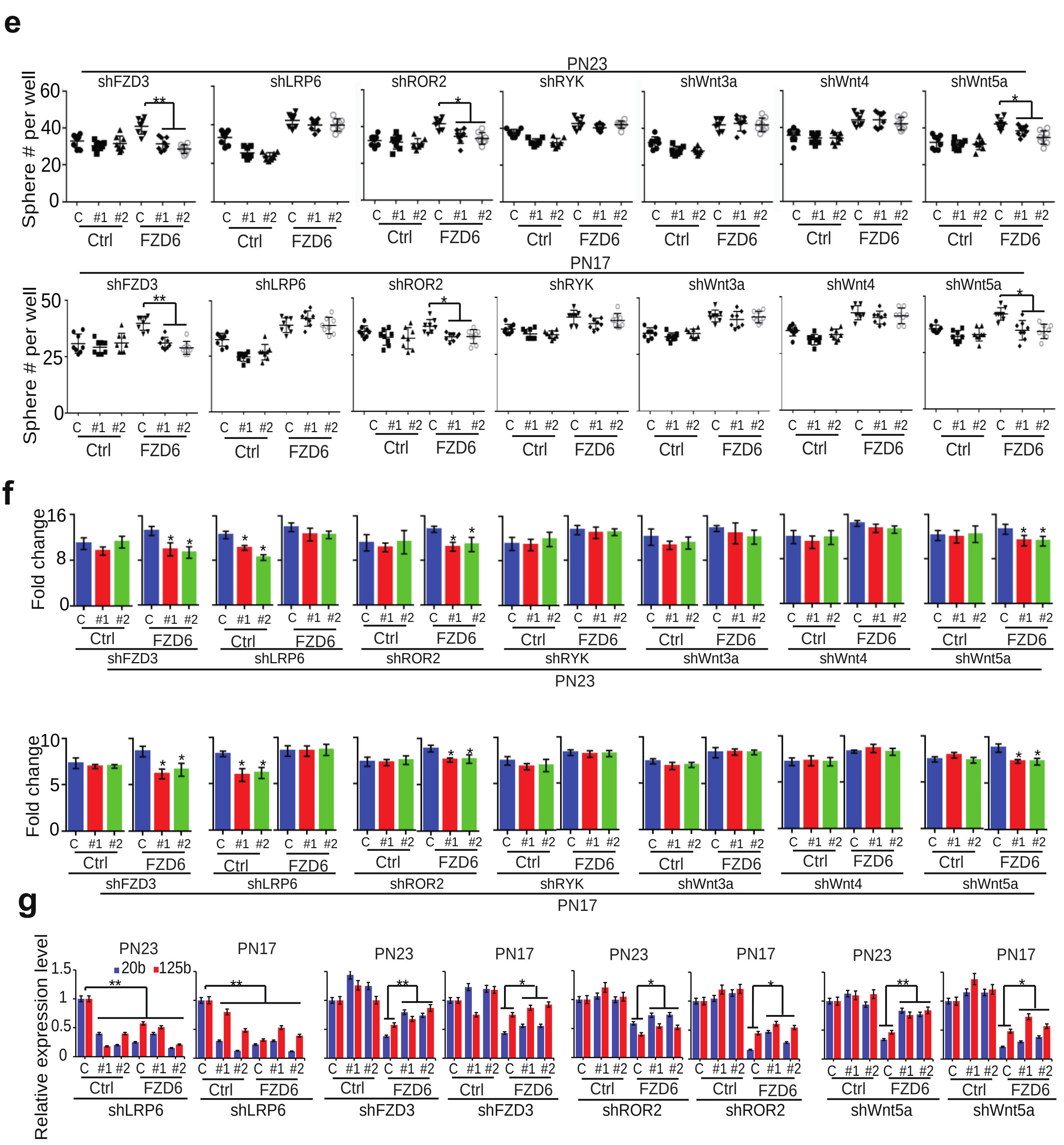

Supplementary Figure 10. (Related to Figure 6) FZD3, ROR2 and Wnt5a are involved in FZD6 overexpression-induced phenotypes in glioma PN spheres. (**a**) IB analysis on PN 23 and 17 spheres with or without FZD1, FZD3 or FZD5 overexpression using indicated antibodies. (**b**) Cell proliferation assays on PN 23 and 17 spheres with or without FZD1, FZD3 or FZD5 overexpression. (**c**) IB analysis using indicated antibodies on PN 23 and 17 expressing FZD6

or a control vector, with or without knockdown of FZD3, LRP6, ROR2, RYK, WNT3a, WNT4 or WNT5a. C: a control shRNA, shRNA #1/#2 are the specific shRNAs of indicated gene. (**d**) Due to incapability of detecting WNT3a and WNT5a using IB with commercial available antibodies, qRT-PCR analyses were used to validate mRNA expression of WNT3a and WNT5a in PN 23 and 17 expressing FZD6 or a control vector with or without knocking down of WNT3a or WNT5a. (**e**) Neurosphere formation assays, and (**f**) cell proliferation were analyzed in indicated glioma spheres. (**g**) qRT-PCR analysis of the relative expression of miR-20b and miR-125b in indicated PN spheres. Error bars (s.d.) represent data of triplicate samples for each set of experiments. . *p <0.05, **p <0.01, paired two-way Student t-test. Data are representative from three independent experiments with similar results.

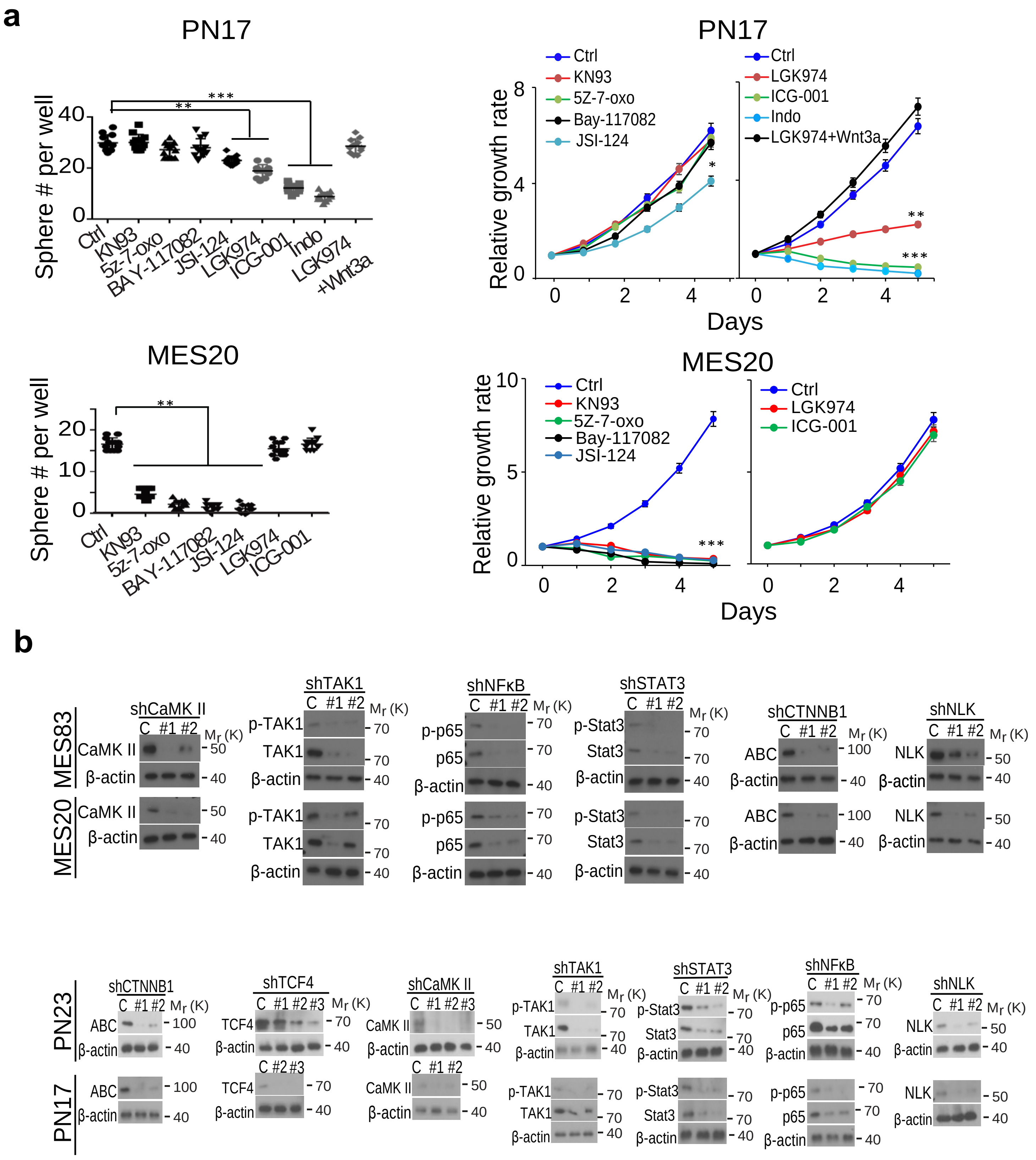
a
PN17
Sphere # per well
Ctrl
KN93
5z-7-oxo
BAY-117082
JSI-124
LGK974
ICG-001
Indo
LGK974 +Wnt3a
PN17
Relative growth rate
Ctrl
KN93
5Z-7-oxo
Bay-117082
JSI-124
Ctrl
LGK974
ICG-001
Indo
LGK974+Wnt3a
Days
MES20
Sphere # per well
Ctrl
KN93
5z-7-oxo
BAY-117082
JSI-124
LGK974
ICG-001
MES20
Relative growth rate
Ctrl
KN93
5Z-7-oxo
Bay-117082
JSI-124
Ctrl
LGK974
ICG-001
Days
b
MES83
MES20
shCaMK II
shTAK1
shNFκB
shSTAT3
shCTNNB1
shNLK
C #1 #2
Mr (K)
CaMK II
β-actin
p-TAK1
TAK1
p-p65
p65
p-Stat3
Stat3
ABC
NLK
PN23
PN17
shTCF4
C #1 #2 #3
TCF4
C #2 #3
shCaMK II

c

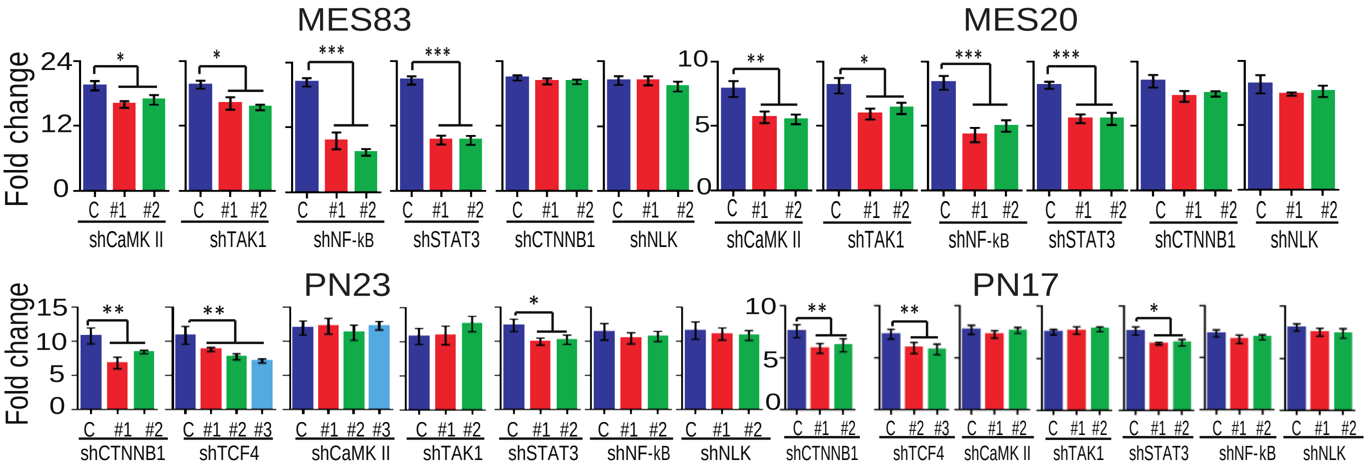
MES83
MES20
Fold change
C #1 #2
shCaMK II
shTAK1
shNF-kB
shSTAT3
shCTNNB1
shNLK
PN23
PN17
shTCF4
C #1 #2 #3
C #2 #3

d

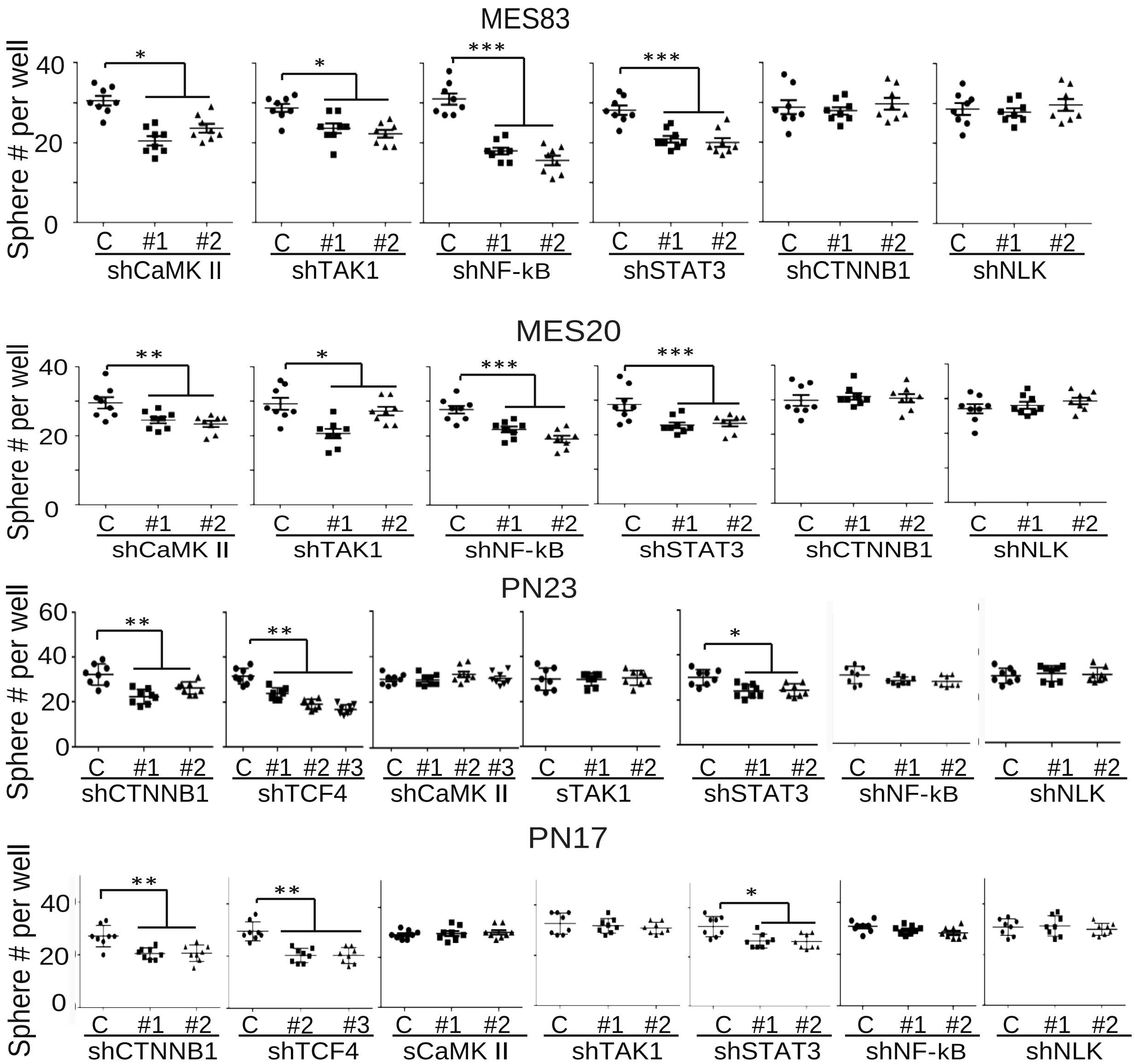
MES83
MES20
PN23
PN17
Sphere # per well
C #1 #2
shCaMK II
shTAK1
shNF-kB
shSTAT3
shCTNNB1
shNLK
shTCF4
sTAK1
sCaMK II

Supplementary Figure 11 (Related to Figure 7) Targeting key components of differentially activated signaling pathways effectively inhibited distinct GBM subtypes. (**a**) Neurosphere formation (left) and cell proliferation (right) of indicated glioma spheres treated with DMSO (Ctrl), KN93 (5 μM), 5Z-7-oxo (5 μM), BAY-117082 (5 μM), JSI-124 (0.1 μM), LGK974 (5 μM), ICG-001 (5 μM) or LGK974 (5 μM) + WNT3a (200 ng/ml), respectively. (**b**) IB analysis using indicated antibodies on MES 83 and 20, PN 23 and 17 glioma spheres expressing control or specific shRNAs targeting CaMKII, β-catenin (CTNNB1), TCF4, TAK1, NF-κB, STAT3, and NLK, respectively. (**c**) Cell proliferation and (**d**) neurosphere formation assays were analyzed on MES 83 and 20, PN 23 and 17 glioma spheres expressing control or specific shRNAs targeting CaMKII, CTNNB1, TCF4, TAK1, NF-κB, STAT3 and NLK, respectively. Error bars (s.d.) represent data of triplicate samples for each set of experiments. *p <0.05, **p <0.01, ***p <0.001, paired two-way Student t-test. Data are representative from three independent experiments with similar results.

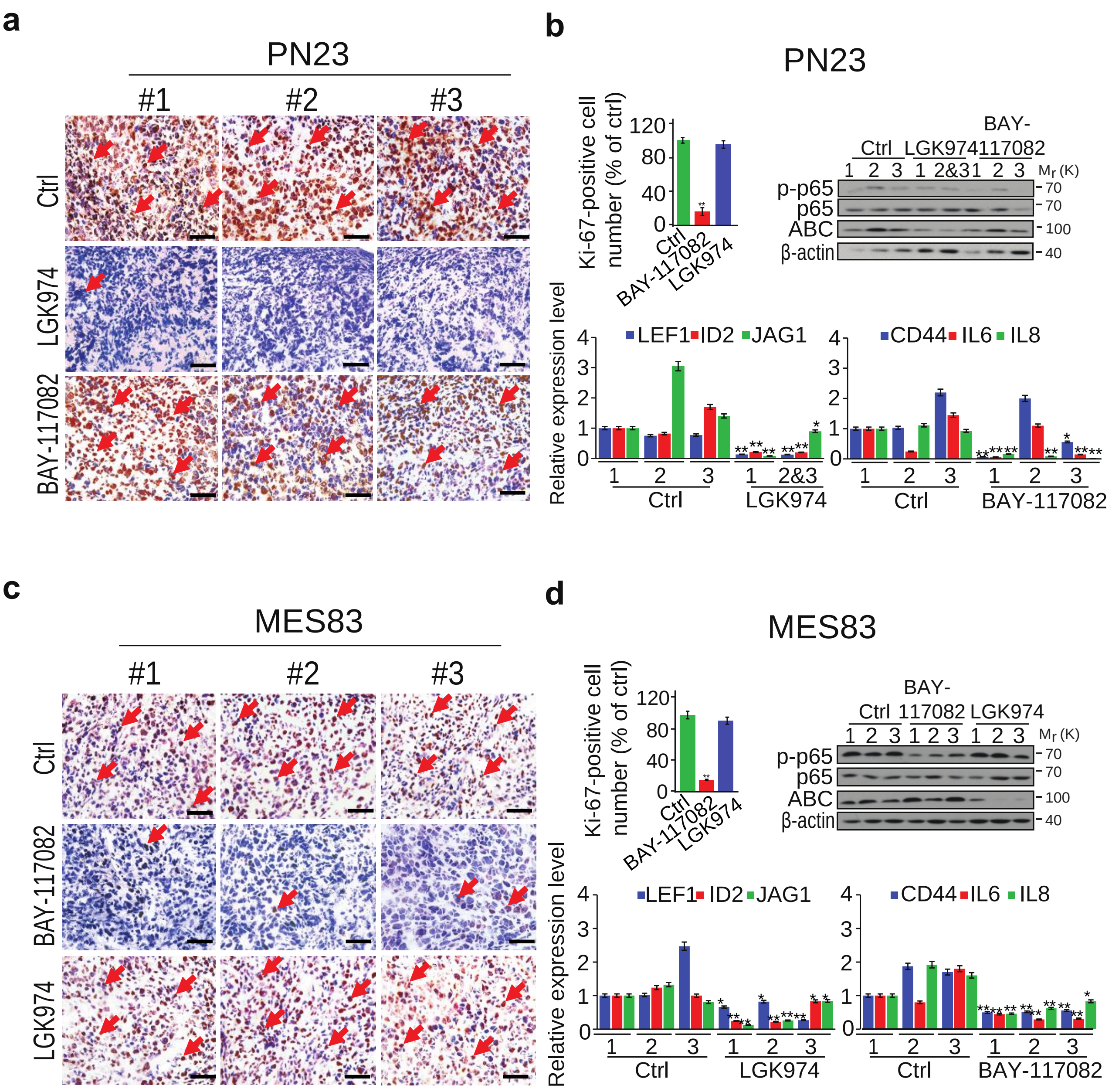

Supplementary Figure 12 (Related to Figure 7) Pathway-specific inhibitors selectively inhibited growth of PN and MES glioma sphere tumor xenografts established by PN 23 (**a,b**) and MES 83 (**c,d**) glioma spheres and Wnt- and NF-κB signaling *in vivo*. (**a,c**), IHC images of Ki-67 stained individual PN 23 or MES 83 glioma sphere tumor xenografts. Scale bar, 50 µm. (**b,d**), Upper left, quantification of Ki-67-positive cells up right, IB of p-p65, p65 and activated β-catenin (ABC) in tumor lysates, lower graphs, qRT-PCR analysis of Wnt pathway target genes, LEF1, ID2 and JAG1 and NF-κB target genes CD44, IL6 and IL8, respectively. In PN GBM tumors, Wnt inhibitor LGK974, but not NF-κB inhibitor BAY-117082, suppressed tumor growth and Wnt signaling. Conversely, in MES GBM tumors, NF-κB inhibitor BAY-117082, but not Wnt inhibitor LGK974, suppressed tumor growth and NF-κB signaling. Data in bar graphs in **b** and **d** were collected from three separate tumors per group and five images per tumor. Error bars (s.d.) represent data of triplicate samples for each set of experiments. *p <0.05, **p <0.01, ***p <0.001, paired two-way Student t-test. Data are representative from three independent experiments with similar results.

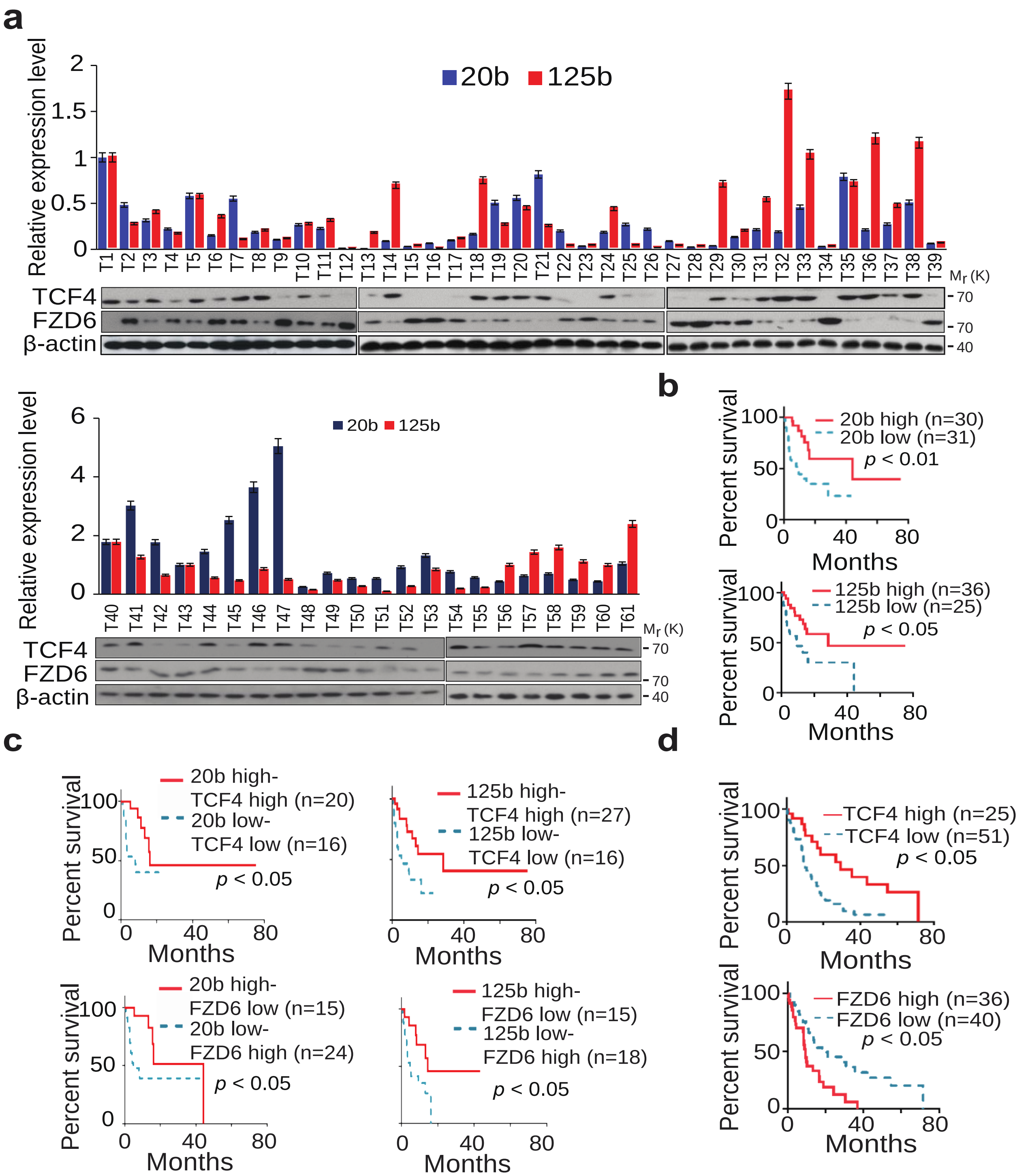

Supplementary Figure 13 (Related to Figure 8) Clinical correlations of expression levels of miR-20b, miR-125b, and TCF4 or FZD6 in clinical GBM tumors. (**a**) qPCR (upper bar graphs) for miR-20b and miR-125b, and IB analysis of 61 snap-frozen clinical GBM tumor specimens using indicated antibodies for expression of TCF4 and FZD6 (lower panels). (**b, c,**) Kaplan-Meier analyses of 61 snap-frozen GBM samples for expression of miR-20b or miR-125b (**b**), in combination of miR-20b or miR-125b with TCF4 or FZD6 (**c**). (**d**) Kaplan-Meier analyses of 76 paraffin-embedded GBM samples analyzed by IHC (see Figure 8c,d) for expression of TCF4 or FZD6. Error bars (s.d.) represent data of triplicate samples for each set of experiments. *p* values were calculated by using log-rank and Gehan-Breslow-Wilcoxon tests.

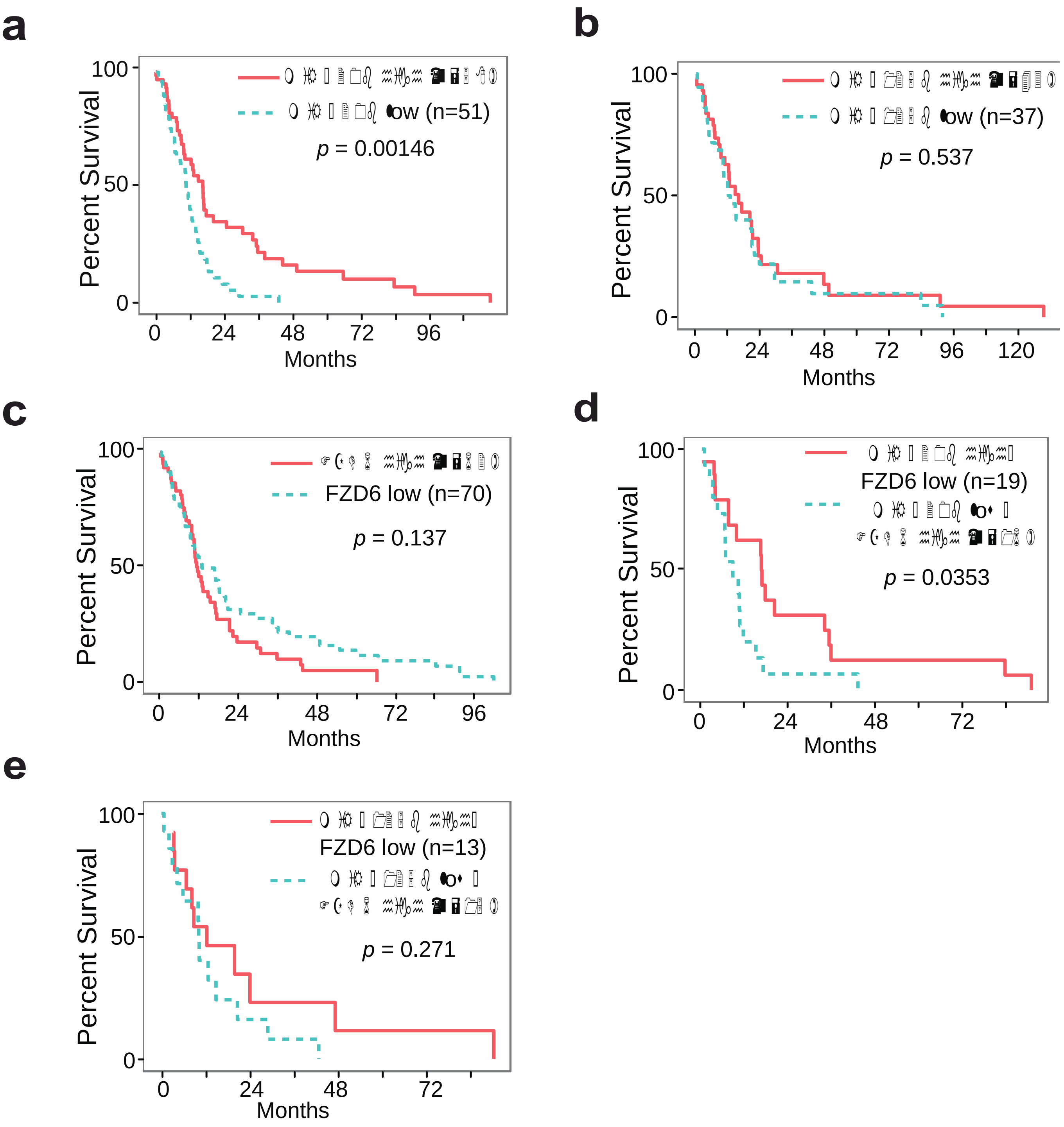

Supplementary Figure 14 (Related to Figure 8e, 8f) Clinical correlations of expression levels of miR-20b, miR-125b and FZD6 in PN and MES GBM tumors available in TCGA data sets. (**a** to **e**) Kaplan-Meier curves of PN and MES GBM tumors available in TCGA data sets were analyzed individually with miR-20b, miR-125b, FZD6, or in combination of miR-20b or miR-125b with FZD6. p values were calculated by using log-rank and Gehan-Breslow-Wilcoxon tests.

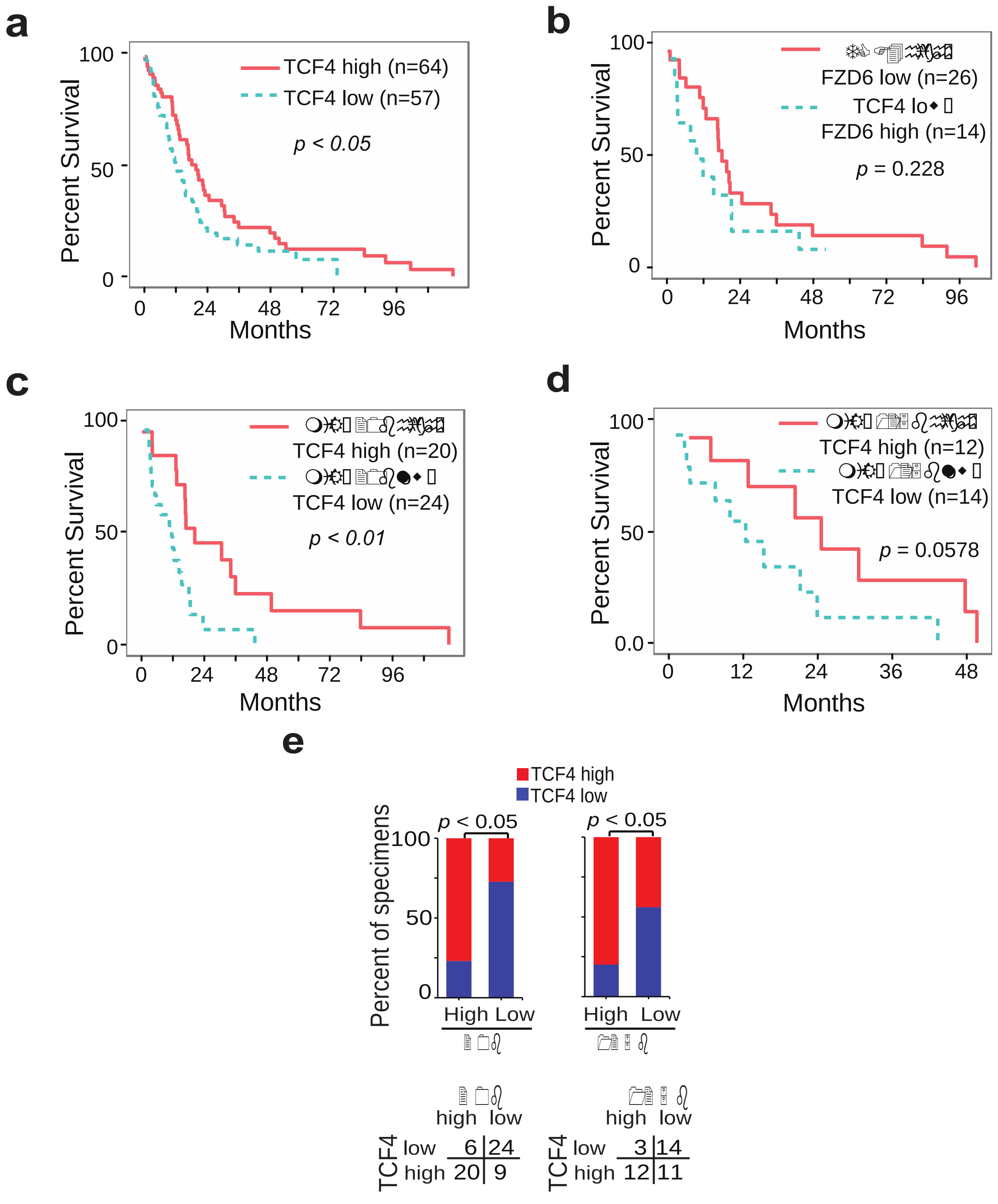

Supplementary Figure 15 (Related to Figure 8e, 8f) Clinical correlations of expression levels of miR-20b, miR-125b and TCF4 in in PN and MES GBM tumors available in TCGA data sets. (**a** to **d**) Kaplan-Meier curves of PN and MES GBM tumors available in TCGA data sets were analyzed individually with miR-20b, miR-125b, TCF4, or in combination of miR-20b or miR-125b with TCF4. (**e**) Percentages of specimens showing high or low miR-20b or miR-125b expression relative to level of TCF4 in TCGA data sets. p values were calculated by using log-rank and Gehan-Breslow-Wilcoxon tests.

Figure 3d

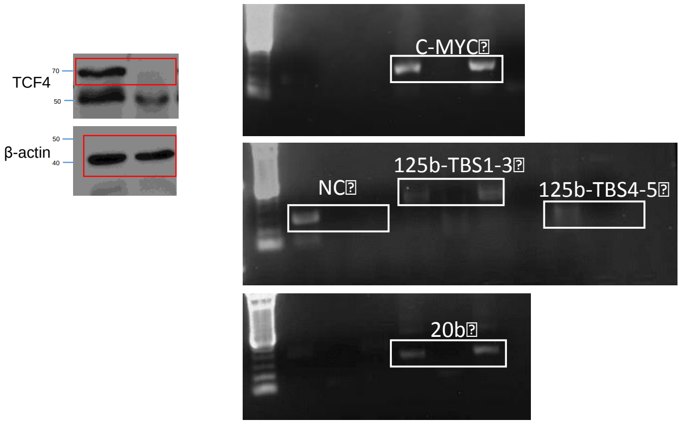

Figure 3g

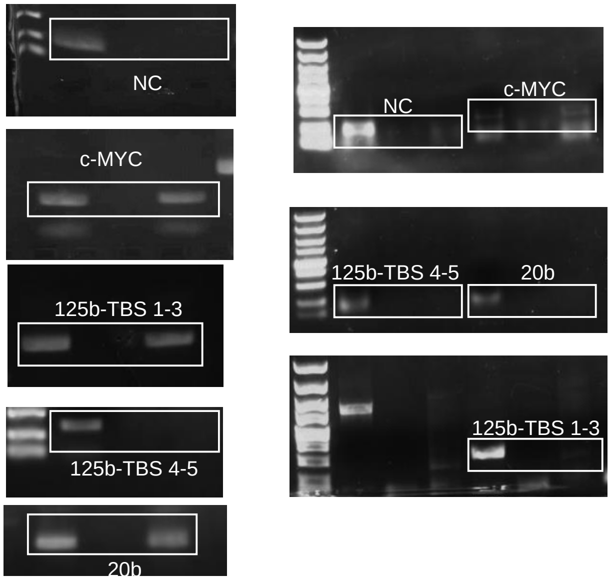

Figure 4c

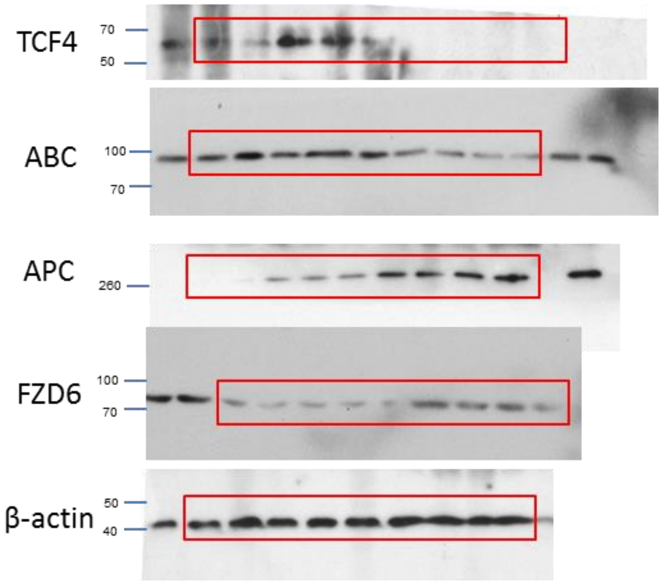

Figure 5a

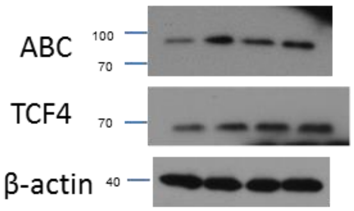

Figure 5d

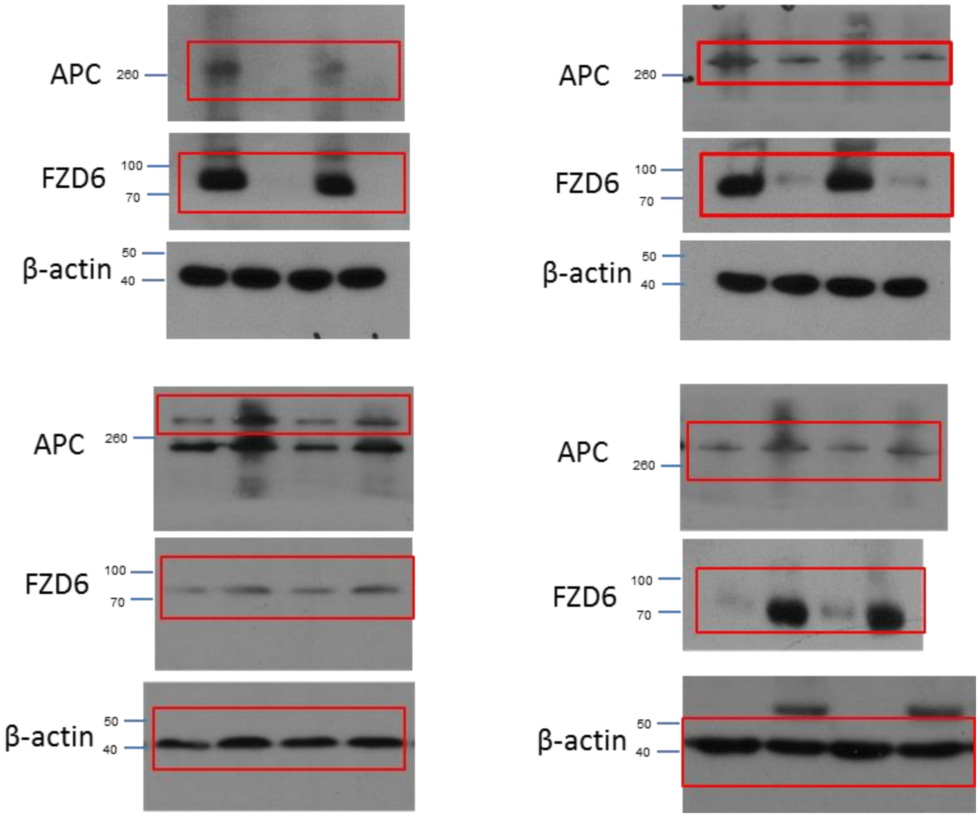

Figure 5e

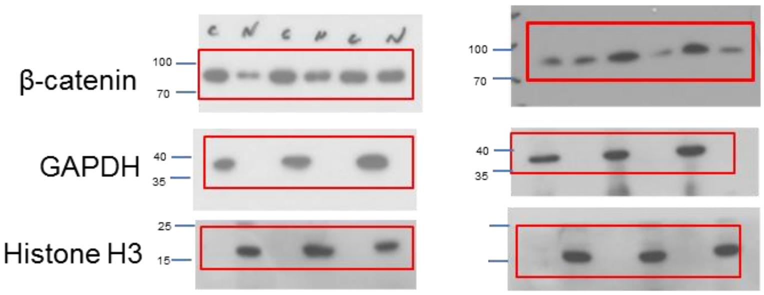

Figure 5g

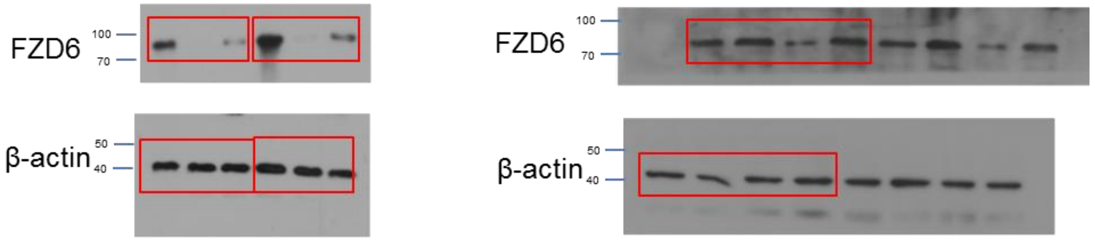

Figure 6a

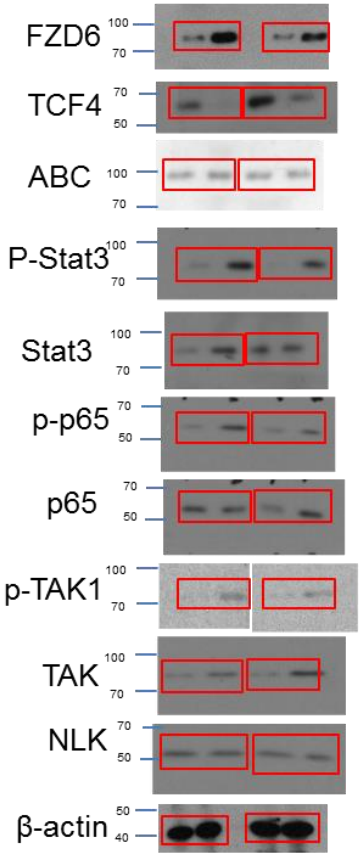

Supplementary Figure 16: Uncropped scans of the IB data presented in the main text (Fig. 3 to 6).

Supplementary Table 1

The list of primers used in this study

S.No.	Gene	Primer	Primer sequence
**Quantitative Reverse Transcription (qRT) primers**			
1	*ALDH1A3*	F	GCATGAGCCCATTGGTGTCT
		R	CGCAGGCTTCAGGACCAT
2	*APC*	F	AAGCATGAAACCGGCTCACAT
		R	CATTCGTGTAGTTGAACCCTGA
3	*APC* (3'UTR)	F	AAGAGAGGAAGAATGAAACTAAGAAAATTC
		R	GCAAAGTGCTGATTATACTTCACTGCTG
4	*FZD6*	F	AGAGGTGAAAGCGGACGGA
		R	AGAGAGTCTGGAGATGGATGCT
5	*FZD6* (UTR)	F	AAACTCGAGTGGGAGGACAGAGTTAGAGGAA
		R	AAAGCGGCCGCTCAGTGTAAAAGCTACCAAAGTGC
6	*HMGA2*	F	TCCCTCTAAAGCAGCTCAAAA
		R	ACTTGTTGTGGCCATTTCCT
7	*ID2*	F	GCTATACAACATGAACGACTGCT
		R	AATAGTGGGATGCGAGTCCAG
8	*JAG1*	F	GGGGCAACACCTTCAACCTC
		R	CCAGGCGAAACTGAAAGGC
9	*LEF1*	F	ATGTCAACTCCAAACAAGGCA
		R	CCCGGAGACAAGGGATAAAAAGT
10	*Olig2*	F	CTCCTCAAATCGCATCCAGA
		R	AGAAAAAGGTCATCGGGCTC
11	*pri-miR-125b*	R	AGAAAAAGGTCATCGGGCTC
		F1	CCATACCACCTGTTTGTTGCATCT
12	*pri-miR-125b*	R1	CTGAGAGGAGCGCAACAATGT
		F2	GAAGAATTCTACCGCATCAAACCA
		R2	CTGCAGACAATCAATAAGGTCCAA
13	*SOX2*	F	CCCTGCTGAGAATAGGACAT
		R	CCCTGCAGTACAACTCTATG
14	*TCF4*	F	TCCCACCACATCATACGCTACAC
		R	TCGCTTGCTCTTCTCTGGACAG
15	*AXIN2*	F	TACACTCCTTATTGGGCGATCA
		R	TTGGCTACTCGTAAAGTTTTGGT
16	*WNT1*	F	CGGCGTTTATCTTCGCTATCA

		R	GCAGGATTCGATGGAACCTTCT
17	*WNT10A*	F	GGAGACTCGCAACAAGATCCC
		R	CGATGGCGTAGGCAAAAGC
18	*WNT10B*	F	GTGAGCGAGACCCCACTATG
		R	CACTCTGTAACCTTGCACTCATC
19	*WNT11*	F	GACCTCAAGACCCGATACCTG
		R	TAGACGAGTTCCGAGTCCTTC
20	*WNT16*	F	GCAGAGAATGCAACCGTACAT
		R	CACATGGGTGTTGTAACCTCG
21	*WNT2*	F	GCCTTTGTTTATGCCATCTCCT
		R	CTTGGCGCTTCCCATCTTCTT
22	*WNT2B*	F	CGGGACCACACCGTCTTTG
		R	GCGAGTAATAGCGTGGACTAC
23	*WNT3*	F	GGAGAGGGACCTGGTCTACTA
		R	CTTGTGCCAAAGGAACCCGT
24	*WNT3A*	F	AGCTACCCGATCTGGTGGTC
		R	CAAACTCGATGTCCTCGCTAC
25	*WNT4*	F	GTACGCCATCTCTTCGGCAG
		R	GCGATGTTGTCAGAGCATCCT
26	*WNT5A*	F	TCGACTATGGCTACCGCTTTG
		R	CACTCTCGTAGGAGCCCTTG
27	*WNT5B*	F	CGCTTCGCCAAGGAGTTTG
		R	TGCCATCTTATACACAGCCCT
28	*WNT6*	F	GGTGCGAGAGTGCCAGTTC
		R	CGTCTCCCGAATGTCCTGTT
29	*WNT7A*	F	CTGTGGCTGCGACAAAGAGAA
		R	GCCGTGGCACTTACATTCC
30	*WNT7B*	F	CGCAGCTATCAGAAGCCCAT
		R	CAGGTGTTGCACTTGACGA
31	*WNT8A*	F	GAACTGCCCTGAAAATGCTCT
		R	TCGAAGTCACCCATGCTACAG
32	*WNT8B*	F	AAGGCCGAGAGTGCCTAAG
		R	CTGCGCGGCTACAGAAGTA

33	*WNT9A*	F	CCACCGTGAGAAGAACTGC
		R	GCCTGCACTCCACATAGCA
34	*WNT9B*	F	TGTGCGGTGACAACCTCAAG
		R	ACAGGAGCCTGATACGCCAT
35	*FZD1*	F	GGGGCTTAACAACGTGGAC
		R	CAGAAAGGACGTGCCGATAAA
36	*FZD2*	F	GTGCCATCCTATCTCAGCTACA
		R	CTGCATGTCTACCAAGTACGTG
37	*FZD3*	F	AATATGGACGTGTCACACTTCC
		R	GGATATGGCTCATCACAATCTGG
38	*FZD4*	F	GTGTCACTCTGTGGGAACCAA
		R	GGCTGTATAAGCCAGCATCAT
39	*FZD5*	F	CCGTTCGTGTGCAAGTGTC
		R	GAAGCGTTCCATGTCGATGAG
40	*FZD7*	F	CAGACGTGCAAGAGCTATGC
		R	ACGATCATGGTCATCAGGTACT
41	*FZD8*	F	ATCTTGTCGCTCACATGGTTC
		R	CATGGTGCCGATGAAGAGGTA
42	*FZD9*	F	TGCGAGAACCCCGAGAAGT
		R	GGGACCAGAACACCTCGAC
43	*FZD10*	F	GCTCATGGTGCGTATCGGG
		R	GAGGCGTTCGTAAAAGTAGCA
44	*LRP6*	F	ACGATTGTAGTTGGAGGCTTG
		R	ATGGCTTCTTCGCTGACATCA
45	*ROR2*	F	ATGGTTCACGACTGCGAATCC
		R	AATGGTCTTCATCCCGTTGGT
46	*RYK*	F	CCCAGGTCAACATTTCTGTTCA
		R	TGCCAGTACAGGAAAGCTCTAC
47	*pri-miR-125b*	R1	CTGAGAGGAGCGCAACAATGT
		F2	GAAGAATTCTACCGCATCAAACCA
		R2	CTGCAGACAATCAATAAGGTCCAA
48	*SOX2*	F	CCCTGCTGAGAATAGGACAT
		R	CCCTGCAGTACAACTCTATG
49	*TCF4*	F	TCCCACCACATCATACGCTACAC
		R	TCGCTTGCTCTTCTCTGGACAG

**Cloning primers**			
1	*FZD6* (ORF)	EcoRI, F	GTTGTTGAATTCGCCACCATGGAGATGTTTACATTTTTGTTGACGTGTA
		NotI, R	GTTGTTGCGGCCGCTCAAGTATCTGAATGACAACCACCTCCC
2	*MIR-20b* promoter	F	GTTTTCGCTTTGGCGGGGTGGG
		R	GCCCCAACGAAGGGCTCCCTTCTTGCC
3	*NLK*	EcoR1	GTTGTTGAATTCGCCACCATGGCGGCTTACAATGGCGGTACATC
		BamH1	GTTGTTGGATCCTCACTCCCACACCAGAGGAGATGGG
4	*TAK1*	EcoR1	GTTGTTGAATTCGCCACCATGTCTACAGCCTCTGCCGCC
		BamH1	GTTGTTGGATCCTCATGAAGTGCCTTGTCGTTTCTGC
5	*FZD1* (ORF)	Xho I, F	GATCTCGAGATGGCTGAGGAGGAGGCGCCTAAGAAGTC
		BamH I, R	ACGGGATCCTCAGACTGTAGTCTCCCCTTGTTTG
6	*FZD3 (ORF)*	EcoR1,F	GTTGTTGAATTCGCCACCATGGCTATGACTTGGATTGTCTTCTCTC
		EcoR1,R	GTTGTTGAATTCGCCACCTTAAGCACTGGTTCCATCTTCTTC
7	*FZD5 (ORF)*	EcoR1,F	AAAAGAATTCATGGCTCGGCCTGACCCATCCGCG
		BamH I,R	ACGGGATCCCTACACGTGCGACAGGGACACCTGCT
**CHIP-sequencing primers**			
1	*mIR-20b* promoter	F	GACAGCGCTCTGTAGAATAAAATG
		R	GTATATAGCCCGGTTGTCTCTCAT
2	*mIR-125b* promoter,1-3	F	AGAAGAACAAGAAGAAGAAAG
		R	TCTCGAGACTGTAACTCTGTAGCTT
3	*mIR-125b* promoter, 4-5	F	TCGAATGGGTGAGTTCAGAACGC
		R	CGCATATACAATCACGCACATACAC
	mIR-MYC promoter	F	GCACGGAAGTAATACTCCTCTCCTC
		R	CAGAAGAGACAAATCCCCTTTGCGC

Supplementary Reference

1. Mao P*, et al.* Mesenchymal glioma stem cells are maintained by activated glycolytic metabolism involving aldehyde dehydrogenase 1A3. *Proc Natl Acad Sci U S A* **110**, 8644-8649 (2013).
